# Effects of growth rate and promoter activity on single-cell protein expression

**DOI:** 10.1038/s41598-017-05871-3

**Published:** 2017-07-24

**Authors:** Niclas Nordholt, Johan van Heerden, Remco Kort, Frank J. Bruggeman

**Affiliations:** 1 Systems Bioinformatics, Amsterdam Institute for Molecules, Medicines and Systems (AIMMS), VU Amsterdam, De Boelelaan 1087, NL1081 HV Amsterdam, The Netherlands; 2 Molecular Cell Physiology, AIMMS, VU Amsterdam, De Boelelaan 1087, NL1081 HV Amsterdam, The Netherlands

## Abstract

Protein expression in a single cell depends on its global physiological state. Moreover, genetically-identical cells exhibit variability (noise) in protein expression, arising from the stochastic nature of biochemical processes, cell growth and division. While it is well understood how cellular growth rate influences mean protein expression, little is known about the relationship between growth rate and noise in protein expression. Here we quantify this relationship in *Bacillus subtilis* by a novel combination of experiments and theory. We measure the effects of promoter activity and growth rate on the expression of a fluorescent protein in single cells. We disentangle the observed protein expression noise into protein-specific and systemic contributions, using theory and variance decomposition. We find that noise in protein expression depends solely on mean expression levels, regardless of whether expression is set by promoter activity or growth rate, and that noise increases linearly with growth rate. Our results can aid studies of (synthetic) gene circuits of single cells and their condition dependence.

## Introduction

The phenotypic state of a cell is largely determined by its repertoire of expressed proteins. Protein concentration, and its variation across isogenic cells, is dependent on various systemic and protein-specific factors. Protein expression depends for instance on the availability of transcriptional and translational machinery, which is growth-rate dependent and considered part of a ‘global-feedback’ mechanism^1–7^. In addition, it depends on protein-specific properties such as regulatory promoter-sequences, the quality of the ribosome binding site and the stability of transcripts and proteins^8, 9^.

Global feedback on protein expression also has important consequences for the physiology of single cells^10^. Fluctuations in global regulatory mechanisms can for instance lead to phenotypic diversification of populations of isogenic cells^11^. They can cause the co-existence of a stress-sensitive, growing subpopulation and a stress-resistant, hardly-growing subpopulation of ‘persister’ cells^12^. Fluctuations in protein concentration and the growth rate of single cells turn out to have a reverberating relation^13^. Stochasticity is therefore an important aspect of protein expression and the phenotype of a single cell.

Single, isogenic cells vary in protein expression^14, 15^ because of systemic and protein-specific stochastic processes^16–19^. Since cell volume and protein content double during the cell cycle, the average number of (constitutively) expressed transcripts and proteins scales with cell volume during balanced cell growth^20^. Spontaneous fluctuations in reaction rates (e.g. transcription and translation), asymmetric division and uneven protein partitioning during cell division cause individual cells to deviate from this average behaviour^19, 21, 22^. Copy-number and volume scaling causes the heterogeneity in protein copy number, across isogenic cells, to be higher than the heterogeneity in protein concentration^19, 20^.

Many noise sources are systemic and contribute to extrinsic noise^16, 17^. Intrinsic noise, in contrast, refers to protein and gene-specific noise sources such as promoter activity, noise propagation from transcriptional regulators, and degradation of transcripts and proteins^15, 21, 23^. Net protein-expression fluctuations result from extrinsic and intrinsic factors, making noise of protein-expression time and cell-state dependent^21, 24, 25^. Understanding protein expression in single cells therefore requires methods for quantification of the contributions of independent noise factors^14, 16, 17, 19, 21^.

The relationship between protein expression noise and the mean protein expression level, in populations of isogenic cells, turns out be very similar across microbial species and growth conditions. Protein expression noise, defined by the ratio of the variance of protein expression and its squared mean value, decreases with the mean expression level until a constant noise floor is reached^26–28^. This noise floor is generally attributed to systemic, extrinsic noise, but its origins are not fully understood. Data suggest that fluctuations in the concentrations of transcription and translation machinery, or translational burst size, may be involved^29–31^. This noise-vs-mean scaling is found regardless of whether protein expression is quantified as total fluorescence per cell, molecule copy number or concentration^26–28^.

Growth rate is an important determinant of protein expression in single cells, influencing intrinsic as well as extrinsic factors. While we understand its influence on the mean protein concentration^6, 32^, via protein dilution, which is species independent, its influences on the stochasticity of protein expression is however much less explored. A complicating phenomenon is that many microbial cells adjust their transcription and translation machinery with growth rate^6^, and the extent to which this compensates for protein dilution and influences protein expression noise is not well understood, and likely species dependent.

In this study, we exploited a titratable, constitutively-expressed, fluorescent reporter protein to investigate the role of growth rate and promoter activity on protein expression and its cell-to-cell variability, using the bacterium *Bacillus subtilis* as our model organism. Such a protein is very suitable for studying effects of growth rate on protein expression in single cells, as it does not have a catalytic activity that influences growth rate. It serves as a reporter for growth rate effects on protein expression if the promoter activity is monitored at constant transcription inducer concentration and variable growth rates. A comparison of protein expression in single cells at constant growth rate and at variable transcription inducer concentrations shows the effects of promoter activity.

We analyse single-cell fluorescence data, obtained with flow cytometry, within a theoretical framework of protein expression under conditions of balanced growth. In balanced growth, attributes of a whole population such as population volume, biomass and total protein increase at the same rate; as a consequence, the distributions of properties of individual cells, such as cell size and protein concentration, become time invariant^33^. Combining theory and noise decomposition, we disentangle the protein expression noise that we observed in our experiments into contributions from extrinsic, systemic and intrinsic, protein-specific sources. The theory we present is not limited to bacteria, but is applicable to any organism that exhibits balanced cell growth.

## Results

### Influences of promoter activity and growth rate on single-cell protein expression

**Figure 1:**
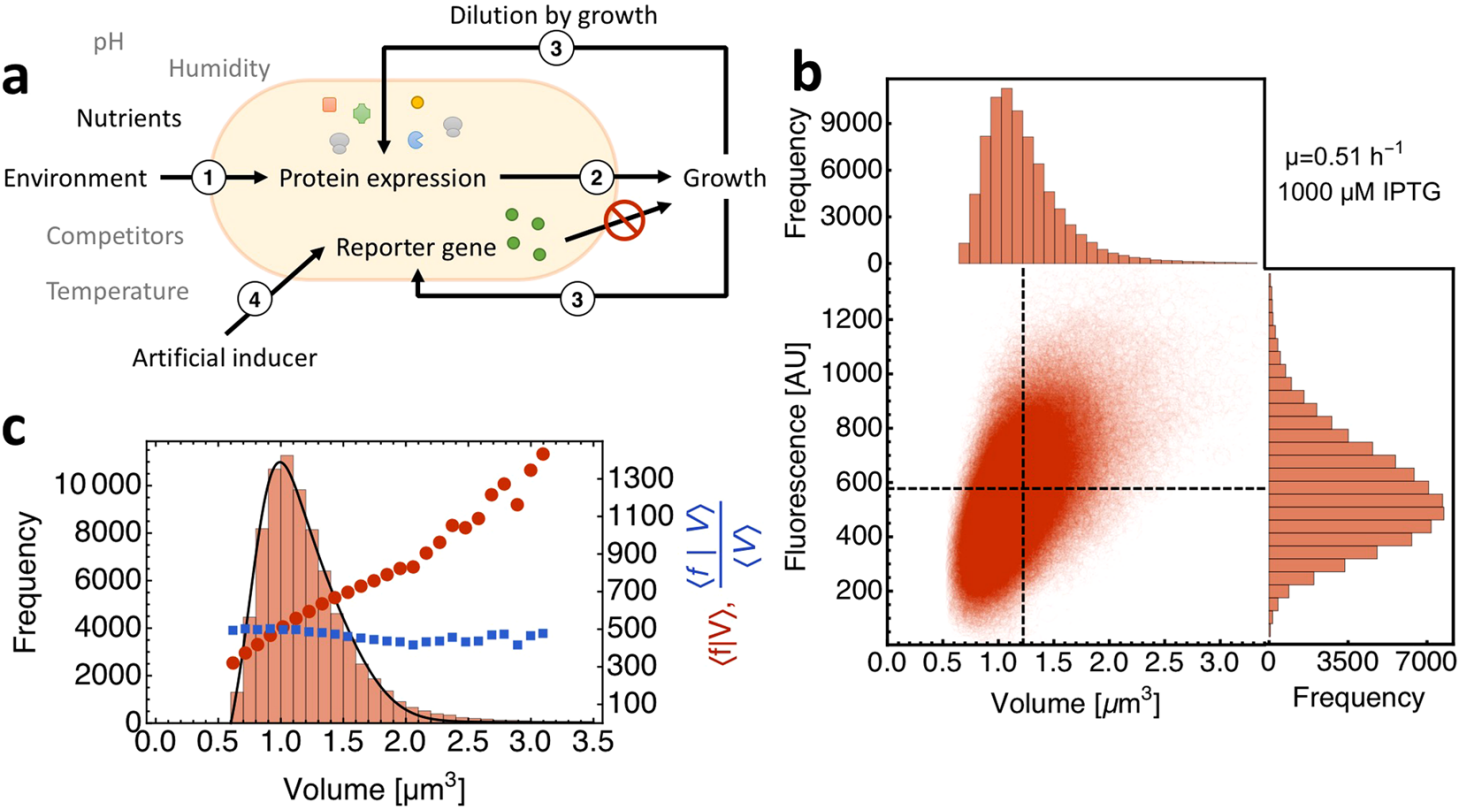
Quantification of the independent effects of growth rate and promoter activity on single-cell protein expression. (**a**) Protein expression in a single cell is set by various factors indicated by the numbered arrows. The environment and cellular control circuits influence protein expression via their influences on transcriptional and translational rates (1). If the protein contributes to growth it exerts an effect on growth rate (2). The resulting physiological state of the cell feeds back onto protein expression (3) via for instance dilution by volume-growth. Usage of a titratable, non-catalytic protein allows for the quantification of growth feedback on protein expression at different promoter activities (4). (**b**) Flow cytometric quantification of single-cell protein expression. For each cell, we measured its total fluorescence and its scatter values, from which we inferred its volume using a cross-calibration of the Coulter counter and the flow cytometer (Fig. S13, details can be found in the ‘Materials and methods’). These data give rise to a distribution of total cell fluorescence, cell volumes and fluorescence concentration, which is analysed in this paper. (**c**) Relationship between the volume, fluorescence and fluorescence concentration of a cell. During balanced growth, cell fluorescence is proportional to volume (red), such that the fluorescence concentration (blue) remains constant across the population and over time. The black line is a fit according to the equation derived by Collins & Richmond^36^ to describe the extant volume distribution of exponentially growing cells.

In order to separate influences of growth rate and promoter activity on protein expression, we introduced the gene encoding green fluorescent protein (GFP), under control of the synthetic isopropyl -D-thiogalactoside (IPTG) inducible hyper-spank promoter^34, 35^, into the genome of *B*. *subtilis*. Since the fluorescent reporter protein does not exert any catalytic activity that impacts cell physiology, it does not influence growth rate, as long as its protein burden remains negligible. Our data show no evidence for a burden (Fig. S1, in the Supplementary Information). Therefore, we have effectively cut the bidirectional influence between the expression of catalytic protein and growth rate; such that only the unidirectional relation from growth rate to protein expression remains (Fig. 1a).

With the genetically-engineered strain we quantified protein expression at balanced growth and modulated growth rate and promoter activity in an independent manner. The growth rate was varied by changing the carbon source in the growth medium. Promoter activity was varied with the IPTG concentration (note: in the text we use promoter activity and IPTG concentration as interchangeable terms). We used flow cytometry to quantify population growth rate, cell volume and the total fluorescence per cell.

**Figure 2:**
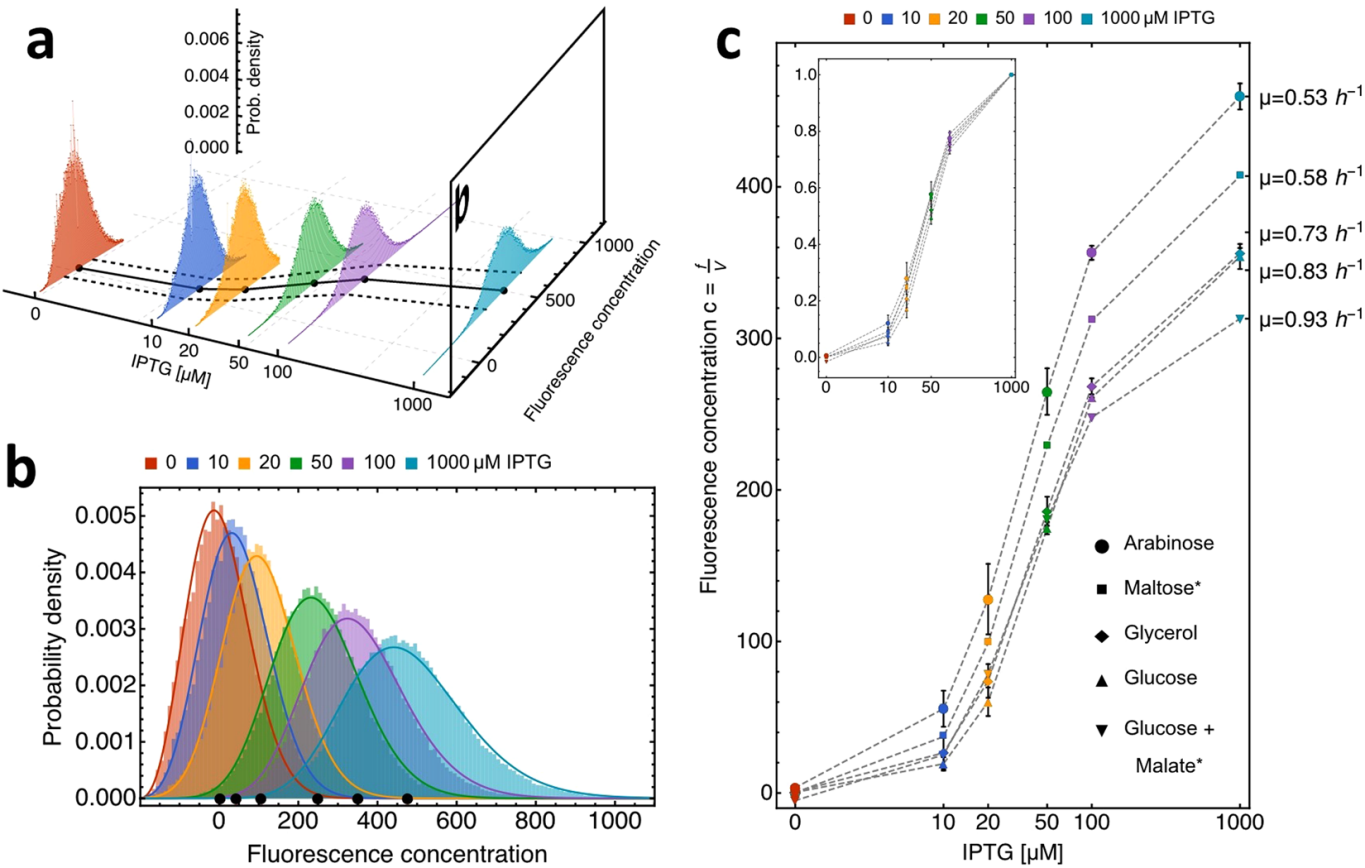
Protein expression is increased by enhanced promoter activity and reduced by enhanced growth rates. (**a**) The distribution of GFP-fluorescence concentration across a population of isogenic *B*. *subtilis* cells, during growth on arabinose (growth rate *μ* ≈ 0.5 h^−1^), as function of the IPTG concentration. The dots indicate mean expression, the line corresponds to the dose-response curve and the dashed lines indicate the mean ± std, indicating cell-to-cell variability. (**b**) The distribution of GFP-fluorescence concentration across a population of isogenic *B*. *subtilis* cells, during growth on arabinose at different IPTG concentration, indicated by the colour of the distributions. Black dots indicate mean values, as in Figure a. Distributions are fitted with gamma distributions (solid lines). (**c**) The mean GFP fluorescence concentration as function of the IPTG concentration at 5 different growth rates, achieved with different carbon sources added to the growth medium. All data points represent the average of two biological replicates, with the exception of maltose and glucose+malate (*), which represent single experiments. Error bars show the standard error of the mean (SEM) of biological replicates. The growth rates indicated on the right side represent the average growth rate for growth on the respective carbon source. The inset shows fluorescence concentration normalised to full induction as function of IPTG, indicating that growth rate influences all protein expression values in the same manner.

Fluorescence per cell and cell volume each follow a positively skewed distribution as described earlier^26, 36^ (Fig. 1b,c). Total fluorescence increases in proportion to cell volume (Fig. 1b). Fluorescence concentration is independent of cell size (Fig. 1c). Both relations are in agreement with the notion that cells grow balanced^33^. (In the inset of Fig. 2c, we normalise the induction curves to their maximal values; they nearly overlap, but not fully, we return to this effect below when we discuss the relation between protein synthesis rate and growth rate.) Other indications of balanced growth are that the number of cells, population volume and fluorescence increased at the same rate for several generation times (Figs S2–S9, in the Supplementary Information). As a result, protein expression (fluorescence concentration) achieved a steady state, for a period longer than several generation times (Fig. S10, in the Supplementary Information). All data that we analyse below correspond to this period of balanced growth.

The influences of growth rate and promoter activity on protein expression are shown in the plots of Fig. 2. As a representative example, data for growth on arabinose (Fig. 2a,b) show that mean protein expression, expressed in fluorescence concentration, increases with the IPTG concentration and that individual cells show variable protein expression. The measured distributions of fluorescence concentration (Fig. 2b) and fluorescence per cell (see Fig. S11, in the Supplementary Information) fit well to gamma distributions, which is in agreement with earlier findings^15, 37–39^. The relation between protein expression and the IPTG concentration is sigmoidal for all carbon sources (Fig. 2c) and protein expression is reduced at higher growth rates. The promoter we used proved very sensitive in a range from 10 to 50 *μ*M IPTG. Within the range of IPTG concentrations used, we observed a maximal induction of 8- to 17-fold, depending on the growth rate. Half-maximal induction was reached around 50 to 70 *μ*M IPTG in all cases. In the absence of IPTG, we did not detect (leaky) expression from the inserted promoter. With increasing induction the fluorescence concentration distributions get wider (Fig. 2b); we discuss this effect below. Additionally, our data show that the mean cell volume increased with the cellular growth rate (see Fig. S1), which is in agreement with earlier findings^40^.

We note that the simultaneous measurement of the volume (calculated from scatter, see Materials and methods) and fluorescence values per cell allows us to quantify protein expression either in total units fluorescence per cell or in fluorescence concentration per cell. These two different units we shall exploit in the decomposition of protein expression noise.

### Enhanced protein dilution by volume-growth at higher growth rate reduces the mean protein concentration

Our data show that both the growth rate and the IPTG concentration influence protein expression during balanced growth (Fig. 2c). The effect of growth rate becomes apparent when one compares protein expression at fixed IPTG concentrations. A higher growth rate reduces protein expression. To understand this effect it is instructive to consider the balance for the protein concentration,

1 $$\frac{dc}{dt}=k(i,\mu )-\mu c,$$

with *c* as the protein concentration, *μ* as the growth-rate and *k* as the protein synthesis rate, which is dependent on the IPTG concentration, *i*, and possibly also on growth rate–hence the notation *k*(*i*, *μ*). Here we consider dilution by volume-growth as the only process that reduces the protein concentration. This is warranted because the GFP which we used is highly stable and not subject to proteolysis^41^.

The protein synthesis rate *k* corresponds to the effective rate of all processes from transcription to the mature protein, many of these are known to increase, to varying degrees, with growth rate^1, 6^. Since we carry out all experiments at balanced growth, the protein concentration is at steady state: $\tfrac{dc}{dt}=0$. We can therefore calculate the protein synthesis rate, by rearranging equation 1, from the product of the growth rate and fluorescence concentration value.

**Figure 3:**
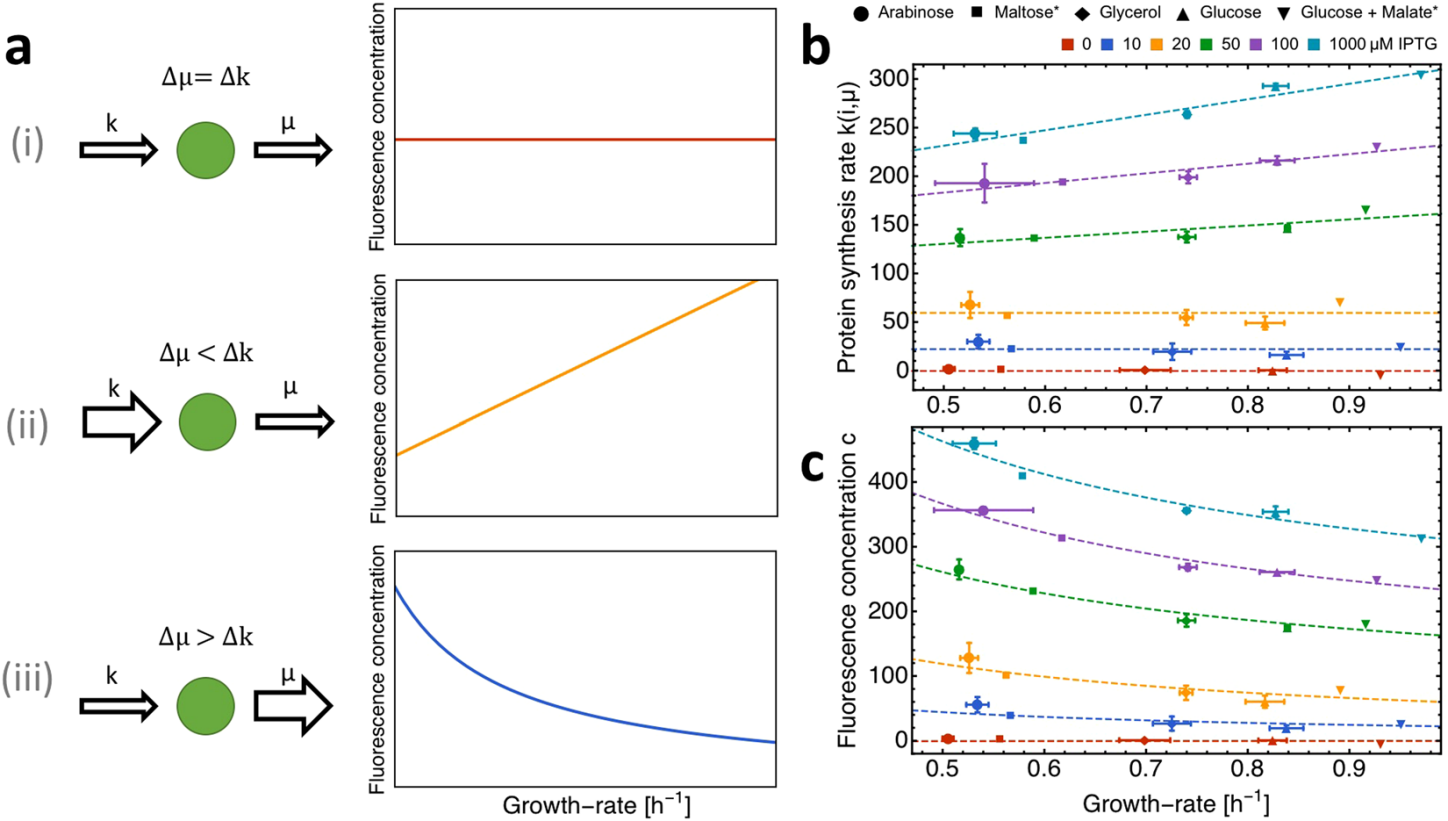
Reduced protein expression by dilution by growth is partially compensated for by enhanced protein synthesis rate at higher growth rates. (**a**) Three scenarios can be distinguished for the response of protein synthesis rate to an enhanced growth rate: (i) perfect compensation, (ii) overcompensation and (iii) undercompensation. (**b**) Protein synthesis rate *k* is invariant to growth rate at low IPTG concentrations and increases linearly with growth rate at intermediate to high IPTG concentrations. Dashed lines are linear fits to the data. (**c**) The change in protein concentration as function of growth rate is dominated by dilution by growth. At IPTG concentrations above 50 *μ*M, an increased protein synthesis rate partially compensates dilution by growth. Dashed lines, model predictions from equation 1 using fitted linear functions for *k*. Data points are either single experiments or the average of two biological replicates. Error bars show the standard error of the mean (SEM) of biological replicates. All individual experimental data points can be found in Fig. S15 in the Supplementary Information.

We distinguish three scenarios of the scaling of the protein concentration with growth (Fig. 3a): (i) perfect compensation (*k* changes in proportion to *μ*), (ii) overcompensation (*k* changes exceed those of growth rate), and (iii) undercompensation (*k* changes are smaller than those of growth rate).

Figure 3b shows the relation between the inferred protein synthesis rate *k* and the growth rate. While at low induction levels *k* is invariant to growth rate, *k* increased with growth rate at IPTG concentrations above 50 *μ*M (Fig. 3b). For an 87% increase in growth rate, we observed an increase of around 20% in protein synthesis rate at full induction.

With the $k(i,\mu )$ values from Fig. 3b (using the dashed, fit-lines) we can calculate the relation between the fluorescence concentration and growth rate (dashed line in Fig. 3c). The resulting relation agrees very well with the measured data. Figure 3c indicates that fluorescence concentration generally decreases with growth rate, indicating that changes in *k* are smaller than those of growth rate (Fig. 3a, scenario iii). At induction levels above 50 *μ*M, the increase in protein synthesis with growth rate alleviates dilution by growth (Fig. 3b), but cannot fully compensate for it, resulting in a net decrease in fluorescence concentration.

We therefore conclude that the relation between mean protein expression (in fluorescence concentration units) with growth rate indicates the undercompensation scenario (mode iii in Fig. 3a).

### Effects of promoter activity and growth rate on protein expression noise are indistinguishable

We quantified the cell-to-cell variability in protein expression, in order to understand how growth rate and promoter activity influence it. To do so, we used the noise measure^14, 15, 18^. It is defined as the variance in protein expression over the squared mean protein expression. It equals the squared coefficient of variation, and quantifies the relative width of the distribution of protein expression across cells. Larger values indicate higher cell-to-cell variability in protein expression. The advantage of using the noise measure, rather than the coefficient of variation, is that variance of independent random variables is additive, which we will exploit below.

**Figure 4:**
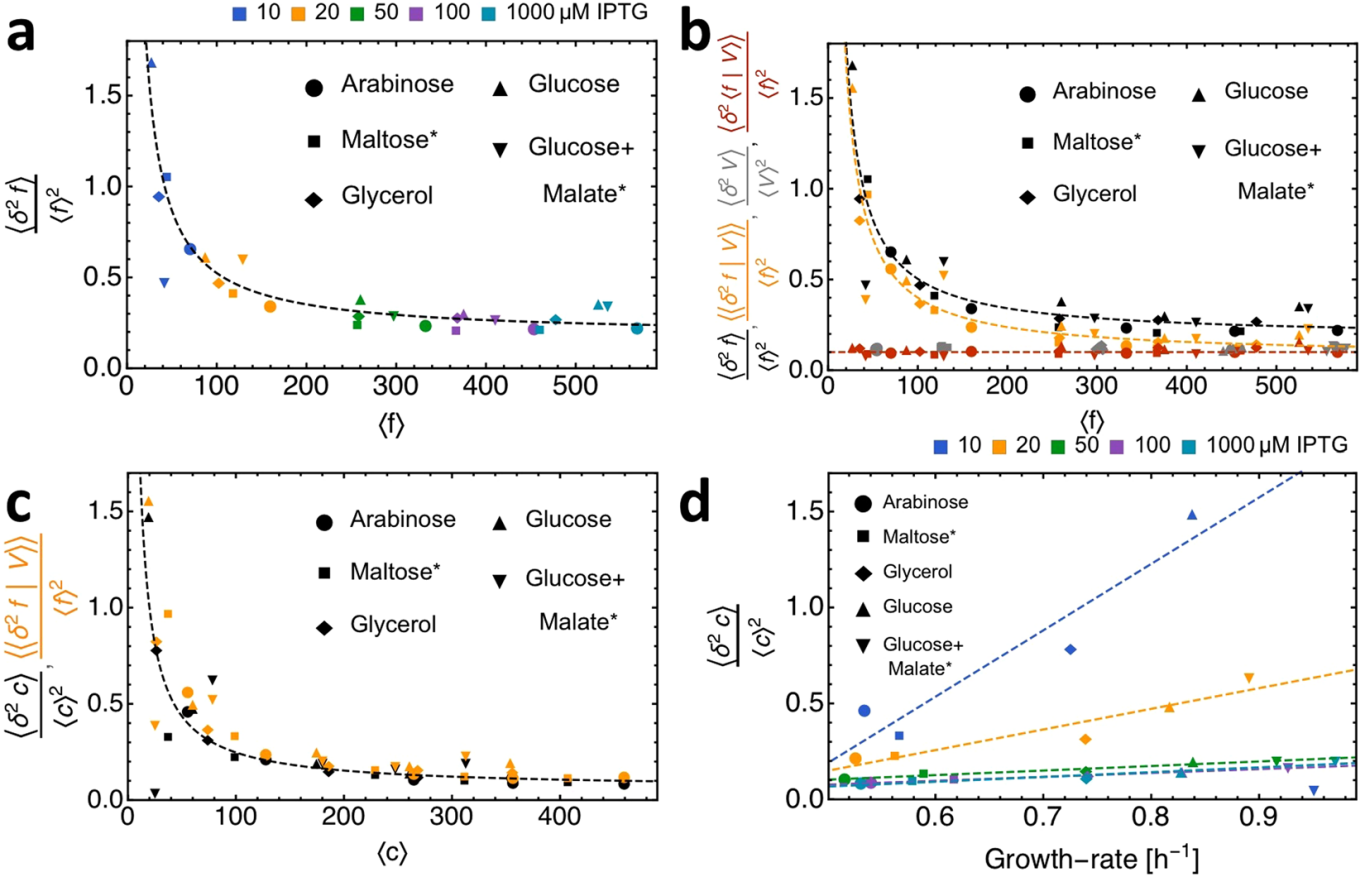
Effects of promoter activity and growth rate on noise are indistinguishable and fall on an invariant relation between noise and mean of protein expression. (**a**) Noise in total cell fluorescence as function of mean total cell fluorescence across growth rates and promoter activities. (**b**) Decomposition of noise in total cell fluorescence into its intrinsic, ‘biochemical’ contribution, $\frac{\langle \langle {\delta }^{2}f|V\rangle \rangle }{{\langle f\rangle }^{2}}$, and its extrinsic, ‘volume-variation’ contribution, $\frac{\langle {\delta }^{2}\langle f|V\rangle \rangle }{{\langle f\rangle }^{2}}$. At balanced growth $\frac{\langle {\delta }^{2}\langle f|V\rangle \rangle }{{\langle f\rangle }^{2}}=\frac{\langle {\delta }^{2}V\rangle }{{\langle V\rangle }^{2}}$, indicating that fluorescence variation scales with volume variation per cell, which is indeed evident from our experimental data. (**c**) Noise in fluorescence concentration per cell is shown as function of the mean concentration. At balanced growth, the following equality holds $\frac{\langle {\delta }^{2}c\rangle }{{\langle c\rangle }^{2}}=\frac{\langle \langle {\delta }^{2}c|V\rangle \rangle }{{\langle c\rangle }^{2}}=\frac{\langle \langle {\delta }^{2}f|V\rangle \rangle }{{\langle f\rangle }^{2}}$, which indicates that protein expression noise due to biochemical origins is directly captured by noise in fluorescence concentration. (**a**–**c**) Dashed lines are fits of the form $\frac{\langle {\delta }^{2}x\rangle }{{\langle x\rangle }^{2}}=\frac{a}{\langle x\rangle }+b$. (**d**) Dependence of noise in fluorescence concentration on the cellular growth rate at different magnitudes of promoter activity. The changes in fluorescence concentration noise can be explained by the decreasing mean expression, through dilution by volume-growth. Dashed lines are linear fits to guide the eye. For each carbon source, data points from 10 to 1000 *μ*M IPTG are shown. Carbon sources with asterisks indicate single experiments, all other data points are the average of two biological replicates. Plots with all individual replicates are shown in Fig. S16, in the Supplementary Information.

We denote the variance and mean of a random variable *X*, with values *x*, by 〈*δ* ^2^ *x*〉_*X*_ and 〈*x*〉_*X*_, respectively; the subscript denotes that we averaged over all values of *X*. The noise value can be calculated from the ratio $\frac{{\langle {\delta }^{2}x\rangle }_{X}}{{\langle x\rangle }_{X}^{2}}$. In Fig. 4a, we plot the relation between the noise in the total cell fluorescence as function of the mean total cell fluorescence values, across growth conditions and promoter activities (i.e. IPTG concentrations), as indicated by different symbols and colours, respectively. Regardless of whether protein expression was changed by growth rate or promoter activities, all data points fall on an invariant noise-vs-mean relation. This characteristic relation holds also for protein concentration (Fig. 4c).

In order to understand how systemic and gene-specific effects contribute to protein expression noise, we decompose the noise in independent terms. We shall denote the mean and variance of *X* values at a particular constant value *y* of another random variable *Y* as ${\langle x|y\rangle }_{X}$ and ${\langle {\delta }^{2}x|y\rangle }_{X}$, respectively. It can now be shown (see SI) that the total variance in *X* equals the sum of two variance contributions: ${\langle {\langle {\delta }^{2}x|y\rangle }_{X}\rangle }_{Y}$ and ${\langle {\delta }^{2}{\langle x|y\rangle }_{X}\rangle }_{Y}$. The term ${\langle {\langle {\delta }^{2}x|y\rangle }_{X}\rangle }_{Y}$ equals the total ‘intrinsic’ contribution. It quantifies the changes in *X* values that occur independently of changes in *Y* values, i.e. changes in *x* at constant *Y* values. The total ‘extrinsic’ contribution ${\langle {\delta }^{2}{\langle x|y\rangle }_{X}\rangle }_{Y}$ quantifies the changes in *X* values due to changes in *Y* values. In the equations below we omit the subscript notation to simplify the notation. For a visual representation of the law of total variance that we exploit here see Fig. S12 in the Supplementary Information.

In Fig. 4b, we decompose the noise in total cell fluorescence into its intrinsic and extrinsic components. The extrinsic component quantifies the variation in fluorescence due to the variation in cell volume, since at balanced growth the fluorescence of a cell is proportional to its volume (see Supplementary Information and Fig. 1c), which is captured by the relation $\langle f|V\rangle =\langle c\rangle V$, with 〈*c*〉 as the mean fluorescence concentration per cell. A consequence is that the noise in total cell fluorescence, generated by a fluorescent protein that is constitutively expressed, equals

2 $$\frac{\langle {\delta }^{2}f\rangle }{{\langle f\rangle }^{2}}=\mathop{\underbrace{\frac{\langle \langle {\delta }^{2}f|V\rangle \rangle }{{\langle f\rangle }^{2}}}}\limits_{{\rm{Intrinsic}}\,{\rm{noise}}}+\mathop{\underbrace{\frac{\langle {\delta }^{2}\langle f|V\rangle \rangle }{{\langle f\rangle }^{2}}}}\limits_{\begin{array}{c}{\rm{Volume}}\,{\rm{noise}}\\ \mathrm{=\ }\langle {\delta }^{2}V\rangle /{\langle V\rangle }^{2}\end{array}}$$

The derivation of this equation can be found in the Supplementary Information.

We note that our interpretation of extrinsic and intrinsic noise is different from that of others (e.g. refs 14, 16, 17). The mathematical procedure is the same, but we condition the protein expression values on cell volume data (which was also done in Kempe, *et al*.^20^); most other current work does not do this. We choose for this approach, because we are interested in the origins of noise in protein concentration, whether is either due to variation in cell volume (a systemic, extrinsic effect) or due to variation in protein copy numbers (a gene specific, intrinsic effect). This means that our intrinsic and extrinsic noise values cannot be directly compared to those obtained by, for instance, Elowitz *et al*.^14^.

Our intrinsic noise term captures variations in protein expression that cause deviations from the balanced-growth relation $\langle f|V\rangle =\langle c\rangle V$. Those are stochastic fluctuations that have biochemical and cellular origins. It includes noise sources such as asynchronous activities of biochemical synthesising and degradation reactions, propagation of reaction noise by fluctuating effector molecules and uneven partitioning of molecules during cell division. The intrinsic noise term decreases with the mean total cell fluorescence (Fig. 4b) until a noise floor is reached at high mean concentrations^26, 28^. This noise floor is thought to arise from sources of noise that do not directly scale with volume, such as fluctuations in the concentration of transcription and translation machinery^29, 31^. The reduction of intrinsic noise with the mean fluorescence level follows $\frac{\langle \langle {\delta }^{2}f|V\rangle \rangle }{{\langle f\rangle }^{2}}=\frac{a}{\langle f\rangle }+b$ (those are the dashed lines in Fig. 4b). In principle, transcription or translation bursts can occur, contributing to the *a* or *b* values, but we cannot decide from our data whether this is the case; this would require knowledge of the true total-cell protein copy number rather than total-cell protein fluorescence.

The extrinsic noise term equals volume noise at balanced growth (see the Supplementary Information). It is determined by cellular heterogeneity in volume. It results from differences in cell cycle progression (cells double their volume on average during a cell cycle), asymmetric division of mother cells, variability in interdivision times, noise in cellular growth rate as function of the cell cycle and other processes. The extrinsic noise term is independent of the mean total cell fluorescence (Fig. 4b), indicating that cell volume noise is hardly changing across growth conditions and promoter activity. This is likely explained by the constant variation in cell volume from birth to division (a factor 2), a constant noise of mother and daughter volumes, and a constant dependency of mean cell volume on cell-age (cell-cycle progression)^20^.

According to us, intrinsic noise in fluorescence concentration is an informative measure about functional heterogeneity in isogenic cells. Since cells with the same protein concentration will experience the same biochemical influence of this protein. Those cells are therefore identical with respect to the functional, or physiological, consequences of this protein. Noise in protein concentration is therefore very informative about the functional noise across isogenic cells.

At balanced growth, noise in fluorescence concentration, intrinsic noise in total cell fluorescence and fluorescence concentration are all identical in value (see Supplementary Information).

3 $$\begin{array}{l}\frac{\langle {\delta }^{2}c\rangle }{{\langle c\rangle }^{2}}=\frac{\langle \langle {\delta }^{2}c|V\rangle \rangle }{{\langle c\rangle }^{2}}=\frac{\langle \langle {\delta }^{2}f|V\rangle \rangle }{{\langle f\rangle }^{2}}:={\rm{functional}}\,{\rm{noise}}\,{\rm{in}}\,{\rm{protein}}\,{\rm{expression}}\end{array}$$

The relation between noise in fluorescence concentration and the mean fluorescence concentration is shown in Fig. 4c. Regardless of how the mean protein expression was varied, via the growth rate or promoter activity, its relation to the noise in protein expression remains unaffected, most noticeably at high mean expression values. At low mean expression levels (low induction levels) we find deviations from the relation. One likely explanation for this is that at low induction levels, noise in transcript copy numbers propagates to protein copy numbers^18^. At low induction levels, a higher growth rate increases the noise because: i. a higher rate of division reduces mean transcript levels and increase transcript noise and ii. more frequent transcript partitioning at cell division also increases transcript noise. The effect of growth rate on protein expression noise diminishes at higher induction levels because noise propagation from transcription to translation decreases.

To identify the relation between functional noise in protein expression and cellular growth rate, we plot noise in fluorescence concentration versus growth rate (Fig. 4d). The individual lines relate noise values at constant promoter activity and therefore indicate the effect of growth rate. We see that noise increases linearly with growth rate, regardless of promoter activity. This is explained by the effect of an enhanced growth rate on the mean protein expression, as this is decreased via enhanced concentration dilution by volume-growth. At low induction levels the deviation from the linear relation is largest because of the effects discussed in the previous paragraph.

## Discussion

The magnitude of protein expression in a single cell depends on promoter activity and cellular growth rate, which are both environment-dependent. The growth rate effect is systemic and affects all cellular proteins, whereas promoter activity effects are protein specific. Promoter activity directly tunes transcription rate, resulting in an altered protein synthesis rate. Growth rate has two opposing effects on protein expression. It can reduce it, via protein dilution due to cell volume-growth, and it can increase expression, as the translation machinery often increases in abundance with growth rate. The net outcomes of promoter activity and growth rate variation on protein expression are therefore not self evident. Their joint effect on cell-to-cell variation in protein expression is even harder to predict. In this paper, we performed experiments to quantify those effects.

We found that promoter activity had the expected effect on protein expression, with a sigmoidal dependence of protein expression on the concentration of the promoter-activity regulator, IPTG. We quantified the dual effect of growth rate on protein expression, i.e. its effect on protein synthesis and degradation, by measuring mean protein expression and cellular growth rate and their product equals protein synthesis rate (when growth is balanced). Mean protein expression decreased as function of growth rate, indicating that protein dilution dominates over growth-rate effects on protein synthesis. Conversely, protein accumulates at lower growth rates, indicating a mechanism of ‘passive regulation’, which was shown recently for the sporulation response in *B*. *subtilis* ^4^. At high expression levels we found that protein synthesis increased with growth rate, which partially compensates for protein dilution.

A striking result is that the relation between the noise and mean of protein expression is nearly independent of how protein expression was changed and regardless of which unit for protein expression is taken, total fluorescence per cell or fluorescence concentration per cell (Fig. 4a,c). At low mean expression levels we observe deviations from this relation; likely due to propagation of transcription and cell-division noise. Promoter activity and growth rate effects therefore fall predominantly on the same noise-vs-mean relation. In addition, noise decreases with mean expression levels and extrapolating the relation suggests the existence of a noise floor at expression levels that are higher than those we measured, which is in agreement with other studies^26–28^. Our data, obtained with a single fluorescent protein, are therefore in agreement with other, broader studies.

Various studies have found that protein expression noise decreases as function of the mean expression level until a noise floor is hit^26–28^; see Sanchez & Golding for a recent review^42^. This ‘universal’ noise-vs-mean relation has been found for a large set of genes. Part of its underlying mechanism must therefore be gene/promoter-sequence independent, often referred to systemic, extrinsic noise^42^. Our data follows the universal relation (Fig. 4b,c). We find it both for total cell fluorescence and fluorescence concentration, which indicates that protein expression noise, when corrected for cell-volume variation (an extrinsic noise component), retains a noise floor. Other extrinsic noise terms, such as fluctuations in transcription and translational machinery, also play a role in the setting the noise floor. This contributes for about 50% in our data (compare Fig. 4b,c, at the maximal fluorescence and concentration values).

A novel insight from our work is that the effects of growth rate and promoter activity on the mean and noise of protein expression are indistinguishable. From the relation between mean and noise that we observe in Fig. 4a we can speculate that in bacteria, noise in constitutive protein expression increases with growth rate because it causes a reduction of mean expression by way of increasing protein dilution by volume-growth. As the noise-vs-mean relation appears universal (see above), we expect the growth rate effect to be universal as well. This statement is not limited to constitutive proteins, since also proteins that have a growth-rate dependent promoter-activity will have fallen on the noise-vs-mean relation. Regulated genes are, however, expected to have elevated noise levels, due to noise propagation from noisy regulators which can be present at low copy numbers. More experimental studies are required to test our expectations.

We illustrate that the units of protein expression, whether expressed in total fluorescence per cell or fluorescence concentration per cell, matter for the quantification of functional noise–noise that has a phenotypic consequence. Noise in concentration captures functional noise^20^. Since noise in total cell fluorescence contains a contribution from the variation of cell volume, its correction for volume noise reveals functional noise, which is illustrated by equation 2 (valid in balanced growth). Our experimental data are in accordance with those equalities (Fig. 4c). Besides indicating how functional noise can be obtained from single-cell fluorescence data, it also provides a consistency check of the fluorescence and cell-volume data.

The consequences of this work are two-fold. From a fundamental perspective this paper indicates that cellular regulation of protein expression, via promoter activity or growth rate influences, impacts protein expression noise via their effect on mean protein expression. As a result, noise of protein expression increases with growth rate because it reduces mean protein expression. This effect could facilitate phenotypic diversification even under optimal conditions, where growth rates are high. From a practical point of view, our work has consequences for the design of synthetic circuits composed out of multiple proteins. Synthetic circuit function is likely to be condition dependent, due to cellular growth rate effects.

## Methods

### Strains and medium composition

For growth experiments, prototrophic *Bacillus subtilis* strain BSB1^43^ was cultivated in a defined morphilinopropanesulphonic acid (MOPS) - buffered minimal medium (MM) containing: 40 mM MOPS (adjusted to pH 7.4), 2 mM potassium phosphate (pH 7.0), 15 mM (NH_4_)_2_SO_4_, 811 *μ*M MgSO_4_, 80 nM MnCl_2_, 5 *μ*M FeCl_3_, 10 nM ZnCl_2_, 30 nM CoCl_2_ and 10 nM CuSO_4_^44^. The medium was supplemented with different carbon sources to the following final concentrations: 5 mM glucose, 10 mM glycerol, 6 mM arabinose, 2.5 mM maltose, 3.75 mM malate and 2.5 mM glucose. From a 1 M stock solution of isopropyl *β*-D-1-thiogalactopyranoside (IPTG) an appropriate amount was added to the medium to reach a final concentration of 0, 10, 20, 50, 100 or 1000 *μ*M.

*Escherichia coli* strain JM109 (Promega) was used for cloning and amplification of plasmids. For cloning, *E*. *coli* and *Bacillus subtilis* were grown in LB + 0.5% w/v glucose supplemented with the appropriate antibiotic in the following concentrations: ampicillin, 100 *μ*g/ml; spectinomycin 150 *μ*g/ml. For LB plates, 1.5% w/v agar were added prior to autoclaving.

Plasmid pDR111-N015-superfolderGFP was constructed by amplifying the coding sequence of superfolderGFP (sfGFP) by PCR with primers N015 (ggtggtgctagcaggaggtgatccagtatgtctaaaggtgaagaactg) and N017 (ggtggtgcatgcttatttgtagagctcatccat), digestion of the product and backbone pDR111^45^ (*bla amyE*’ *spc* ^*R*^ *P*_*hyperspank*_ *lacI*’ *amyE*; kind gift from David Rudner) with NheI and SphI and subsequent ligation. After transformation of chemocompetent *Escherichia coli* JM109 (Promega) and plasmid isolation, the identity of pDR111-N015-sfGFP was confirmed by sequencing. *Bacillus subtilis* strain B15 (BSB1 *spc* ^*R*^ *P*_*hyper*–*spank*_-*sfGFP lacI*::*amyE*) was constructed as following: pDR111-N015-sfGFP was linearised with SacII, added to a BSB1 culture grown in MM+glucose until starvation phase, and incubated for one hour before addition of fresh MM and plating on LB+glucose+spc for selection. Genomic insertion into *amyE* was confirmed by amylase deficiency, PCR and sequencing. The *amyE* locus is situated at ≈28 degrees on the genome. We address the influence of the maturation time of the used fluorescent protein in the SI.

### Growth experiments

Cells were inoculated directly from single-use 15% glycerol stocks into 50 ml Greiner tubes with 5 ml MM supplemented with IPTG and grown at 37 degrees Celcius and 200 rpm. After 10 to 15 generations, the cultures reached an OD between 0.01 and 0.2 and were diluted in 250 ml Erlenmeyer flasks with 50 ml fresh, pre-warmed MM with IPTG to an OD of 0.0001 to 0.0003 and growth was monitored at least twice per generation. At each time-point, 500 *μ*l of culture were sampled into 2 ml eppendorf tubes and immediately subjected to flow cytometry. For each experiment, the wild type BSB1 culture was taken along under the same conditions as the B15 cultures to correct for autofluorescence and control for effects of IPTG or GFP expression on cell growth.

#### Balanced growth criterium

In each experiment, we monitored population volume, fluorescence and cell count for several hours and calculated the specific growth rate for each of these properties (Figs S2–S9). In balanced growth, extensive attributes such as population volume and fluorescence as well as cell number increase exponentially at the same rate, resulting in concentration homeostasis of GFP^33^. For all analyses, we defined a region of at least 1.5 generation times in which fluorescence concentration was most stable (Fig. S10, in the Supplementary Information).

### Flow cytometry

For all experiments, a BD Accuri C6 Flow Cytometer with the manufacturers software was used to acquire counts, fluorescence and light scattering properties of single cells in 20 *μ*l of culture. The software settings were as following: Fluidics, slow; Threshold, 15000 on FSC-H; Run with limits, 20 *μ*l. Undiluted cultures were used up to a cell count of no more than 5000 events per second at which point the samples were diluted 1:10 in pre-warmed MM of the same batch. Fresh MM without cells was used as a background control. After each experiment, all data were exported in FCS format and analysed using MATHEMATICA, version 10 (Wolfram Research, Champaign, IL, USA).

### Cross-calibration of forward scatter area and cell volume

Forward scatter correlates with volume^46^. Comparing the distributions of volume and forward scatter area (FSC-A) of a population of cells indicates a linear relationship between those two properties. To calculate volume from FSC-A, we performed calibration experiments with all 5 carbon sources, using a Beckman Coulter Multisizer 3 Coulter counter for volume determination. Per carbon source, BSB1 cells were grown as described in section 0. After propagation, cells were grown for 5 to 6 hours when samples were taken and subjected directly to flow cytometry or, after 1:200 dilution in 10 ml ISOTON diluent (Beckman Coulter), measured on the Multisizer 3. Dilutions for the Multisizer 3 were done in triplicates and run with identical settings. Bins were centred and counts per bin were averaged over the triplicates. As a sanity check and to control for dilution artefacts, we calculated the number of cells per *μ*l from both data sets (Fig. S13, in the Supplementary Information). To convert FSC-A to volume, we computed every other percentile in a range from the 2nd to the 98th of both, the volume and FSC-A distributions and fitted a linear function through them (Fig. S13, in the Supplementary Information). The linear function was used to convert FSC-A to volume.

### Data analysis

Data analysis was carried out in MATHEMATICA, version 10 (Wolfram Research, Champaign, IL, USA), using custom scripts. As a pre-filtering step, all events with an FSC-H ≤ 18000 and an FSC-A ≥ 150000 were discarded. The first filter was applied to exclude small particles such as cell debris, the second filter to exclude chains of cells and other measurement artefacts. From each file, the beginning time of acquisition was extracted and for each event, FSC-A was converted to volume [*μ*m^3^] and exported along with the GFP fluorescence channel area value (FL1-A; excitation 488 nm, emission 533 nm).

#### Calculation of cellular growth-rate

Growth-rates were calculated based on cell counts at each time point. Particle counts from medium-only samples were subtracted at each time point to correct for background, such as salt precipitate, in the medium. The background was low in all samples and didn’t affect the calculated growth rate. The first and last time point of each experiment were excluded from analysis. Using the MATHEMATICA function LinearModelFit, a linear fit through the Log-transformed data was computed. The slope of this fit gives the specific growth rate *μ* in units [h^−1^]. There were no differences in growth rate between BSB1 and B15 under the same conditions (Fig. S13, in the Supplementary Information).

#### Correction for autofluorescence strength

Autofluorescence can contribute a significant amount to total fluorescence and mask actual GFP signals. Autofluorescence correlates with cell size under identical conditions (data not shown), so we chose a correction method based on cell size. All corrections described here were carried out for all individual carbon sources, IPTG concentrations and time points, using BSB1 wild type cultures that were taken along during all experiments.

Cells were binned by volume into fixed bins of width 0.2 *μ*m^3^ and the mean FL1-A was calculated for BSB1 wild type samples. This mean value was then subtracted from all single events in the corresponding bin of the B15 sample. Bins with less than 20 cells in either of the samples were excluded from further analysis.

#### Correction for autofluorescence variance

The variance in background fluorescence can contribute significantly to variance in total fluorescence at low induction levels (Fig. S14, in the Supplementary Information). This leads to a distortion of the noise measure at low mean fluorescence, such that an apparent scaling of noise with the variance over the mean squared can be observed (Fig. S14, in the Supplementary Information). Correcting for this background variance reveals the inverse proportionality of noise to the mean that has been observed before refs 26, 28, 47.

To estimate the variance in background fluorescence, we corrected the BSB1 wild-type samples for autofluorescence as described above, effectively shifting their mean to 0. We then calculated the volume-conditional variances in fluorescence and fluorescence concentration (see section on noise in the Supplementary Information) at each time point and IPTG concentration in balanced growth and removed data points with the highest and lowest variance for each sample. In the following we make use of the law of total variance (refer to noise section in the Supplementary Information for details). For each carbon source, the mean variance of all time points and IPTG concentrations was used as an estimate for variance of background fluorescence $\langle {\delta }^{2}{f}_{bg}\rangle ={\langle {\langle {\delta }^{2}{f}_{bg}|V\rangle }_{f}\rangle }_{V}+{\langle {\delta }^{2}{\langle {f}_{bg}|V\rangle }_{f}\rangle }_{V}$ or fluorescence concentration $\langle {\delta }^{2}{c}_{bg}\rangle ={\langle {\langle {\delta }^{2}{c}_{bg}|V\rangle }_{c}\rangle }_{V}+{\langle {\delta }^{2}{\langle {c}_{bg}|V\rangle }_{c}\rangle }_{V}$. The variance in fluorescence or fluorescence concentration resulting from GFP was then simply calculated by subtracting 〈*δ* ^2^ *f*_*bg*_〉 and 〈*δ* ^2^ *c*_*bg*_〉 from 〈*δ* ^2^ *f*_*total*_〉 and 〈*δ* ^2^ *c*_*total*_〉, respectively.

For all subsequent analyses, the average of all time points from the region in which fluorescence concentration was stable in time was used (these points are shown in Fig. S10).

### Data availability

The datasets generated during the current study are available from the corresponding author on reasonable request.

## Electronic supplementary material

Supplementary Information
