# Supplementary material for: Effects of growth rate and promoter activity on single-cell protein expression
Source: Sci Rep. 2017 Jul 24;7:6299. doi: 10.1038/s41598-017-05871-3 (PMC5524720; doi:10.1038/s41598-017-05871-3)
Supplement: Supplementary file 1 — Supplementary Information [file 41598_2017_5871_MOESM1_ESM.pdf]

Supplementary Information: Effects of growth rate and promoter activity on single-cell protein expression

N. Nordholt[1], J. van Heerden[1], R. Kort[2], F.J. Bruggeman[1,*]

1. Systems Bioinformatics, Amsterdam Institute for Molecules, Medicines & Systems (AIMMS), VU University, De Boelelaan 1087, NL-1081 HV, Amsterdam, The Netherlands.
2. Molecular Cell Physiology, AIMMS, VU University, De Boelelaan 1087, NL-1081 HV, Amsterdam, The Netherlands.

* corresponding author: f.j.bruggeman@vu.nl

May 17, 2017

Contents

1 Effects of fluorescent protein maturation time **1**

2 Noise theory at balanced cell growth **2**

3 Supplementary figures **6**

1 Effects of fluorescent protein maturation time

To estimate the effect of fluorescent protein maturation time, we consider the following model,

$$\dot{n} = k_p - k_m n - \mu n \quad (1)$$
$$\dot{f} = k_m n - \mu f \quad (2)$$

with f and n as the fluorescent and nonfluorescent protein concentration, k_p as the protein synthesis rate, k_m as the rate constant for maturation and μ as growth rate. This system will reach a steady state with a fluorescent protein fraction $\phi = \frac{f}{n+f} = \frac{k_m}{k_m+\mu}$. The superfolder fluorescent protein which we used has a maturation time of about 10 minutes such that $k_m = 0.1\ min^{-1} = 6\ hr^{-1}$. Given the measured range of growth rates, 0.5-0.9 hr^{-1}, we obtain a range of steady state fractions of $0.87 \leq \phi \leq 0.92$. So we underoverestimate the actual concentration of the fluorescent protein by $\approx 10\%$.

2 Noise theory at balanced cell growth

Concentrations of constitutively expressed proteins remain constant during balanced growth

The rate of change in the concentration c of a molecule with copy number n in a cell with volume V equals

$$\frac{d}{dt}c = \frac{d}{dt}\frac{n}{V} = \frac{1}{V}\frac{d}{dt}n - \frac{n}{V^2}\frac{d}{dt}V \tag{3}$$

When this concentration is constant over time, the cell grows with a constant growth rate, as is indicated by the following relation,

$$\frac{1}{n}\frac{d}{dt}n = \frac{1}{V}\frac{d}{dt}V := \text{ exponential growth rate, } \mu \tag{4}$$

A protein that is therefore constitutively expressed (such that dn/dt is constant) shall at balanced growth, when $d\ln V/dt$ has become fixed for a duration longer than several generation times, display a constant concentration. Our experimental data indeed shows that this condition is met at balanced growth (see figure 10).

A consequence of this balanced growth condition is that at every cell volume the following relation holds, which is indeed in agreement with our experimental data,

$$\langle f|V\rangle = \langle c\rangle V \quad \text{and} \quad \langle f\rangle = \langle c\rangle\langle V\rangle \tag{5}$$

with f as the total fluorescence per cell, which is proportional to the copy number of the fluorescent proteins that it contains.

Short introduction to variance decomposition

We shall first derive the variance decomposition equation. We consider two random variables X and Y, e.g. protein copy number and cell volume, that take on values denoted by x and y (both running from zero to infinity, in principle, and x's are discrete and the y's are continuous). We denote an average of a random variable X as $\langle x\rangle_X$ and its variance as $\langle \delta^2 x\rangle_X$ (the variance equals the mean squared deviation); the subscript denotes that we took an average over X's values, this is useful notation when we consider conditional means, which is what we do next.

We denote the average of a random variable y at a constant value of another (random) variable x – the mean of Y conditioned on a particular value of X – as $\langle y|x\rangle_Y$ and its variance as $\langle \delta^2 y|x\rangle_Y$.

We can rewrite the variance of Y in terms of two contributions using 'variance decomposition', giving rise to the 'law of total variance':

$$\begin{aligned}
\langle \delta^2 y\rangle &= \langle y^2\rangle_Y - \langle y\rangle_Y^2 \\
&= \langle\langle y^2|x\rangle_Y\rangle_X - \langle\langle y|x\rangle_Y\rangle_X^2 \\
&= \langle\langle \delta^2 y|x\rangle_Y + \langle y|x\rangle_Y^2\rangle_X - \langle\langle y|x\rangle_Y\rangle_X^2 \\
&= \langle\langle \delta^2 y|x\rangle_Y\rangle_X + \langle\langle y|x\rangle_Y^2\rangle_X - \langle\langle y|x\rangle_Y\rangle_X^2 \\
&= \langle\langle \delta^2 y|x\rangle_Y\rangle_X + \langle \delta^2\langle y|x\rangle_Y\rangle_X.
\end{aligned} \tag{6}$$

The first term in this equation is the intrinsic contribution and the second term equals the extrinsic contribution. The first term is called intrinsic because it concerns the total variation in Y values when X is constant, so those occurring independent of X; those are intrinsic to Y. Those changes in Y that are due to changes in X can captured by the second terms, those are extrinsic to Y.

Another way to think about variance decomposition is to consider the following relation, which states that y is a function of x plus a noise term that depends on x (visualised in Figure 12),

$$y(x) = f(x) + \epsilon\sigma(x) = \langle y|x\rangle + \underbrace{\epsilon\sqrt{\langle\delta^2 y|x\rangle}}_{\text{noise}}, \ \epsilon \sim N(0,1), \tag{7}$$

with σ as a standard deviation and $N(0,1)$ as a normal distribution with mean 0 and standard deviation 1. We can determine the total variance in y from,

$$\begin{aligned} \langle\delta^2 y(x)\rangle = \langle\delta^2 y\rangle &= \langle\delta^2\langle y|x\rangle + \epsilon\sigma(x)\rangle \\ &= \langle\delta^2\langle y|x\rangle\rangle + \langle\delta^2\epsilon\sigma(x)\rangle \end{aligned} \tag{8}$$

The last term can be simplified further

$$\begin{aligned} \langle\delta^2\epsilon\sigma(x)\rangle &= \langle(\epsilon\sigma(x))^2\rangle - \langle\epsilon\sigma(x)\rangle^2 = \langle(\epsilon\sigma(x))^2\rangle - \langle\delta\epsilon\delta\sigma(x)\rangle - \langle\epsilon\rangle\langle\sigma(x)\rangle \\ &= \langle(\epsilon\sigma(x))^2\rangle = \langle\epsilon^2\sigma(x)^2\rangle \\ &= \langle\delta\epsilon^2\delta\sigma^2(x)\rangle + \langle\epsilon^2\rangle\langle\sigma^2(x)\rangle \\ &= 0 + (\langle\delta^2\epsilon\rangle + \langle\epsilon\rangle^2)\langle\sigma^2(x)\rangle \\ &= \langle\delta^2\epsilon\rangle\langle\sigma^2(x)\rangle = \langle\sigma^2(x)\rangle \\ &= \langle\langle\delta^2 y|x\rangle\rangle \end{aligned} \tag{9}$$

Therefore, equation 8 becomes,

$$\langle\delta^2 y\rangle = \langle\delta^2\langle y|x\rangle\rangle + \langle\langle\delta^2 y|x\rangle\rangle, \tag{10}$$

which is also the equation for variance decomposition (or the law of total variance).

Variance decomposition of noise in total cell fluorescence

The noise in total cell fluorescence is defined by the ratio of its variance and its squared mean value, i.e.

$$\frac{\langle\delta^2 f\rangle}{\langle f\rangle^2} \tag{11}$$

We can determine the role of heterogeneity in cell volume, e.g. due to cell growth, for instance to be able to assess noise in protein concentration later, using variance decomposition (the subscript indicates the random variable for which the mean or variance is calculated):

$$\frac{\langle\delta^2 f\rangle}{\langle f\rangle^2} = \frac{\langle\langle\delta^2 f|V\rangle_f\rangle_V}{\langle f\rangle^2} + \frac{\langle\delta^2\langle f|V\rangle_f\rangle_V}{\langle f\rangle^2} \tag{12}$$

Since $\langle f|V\rangle = \langle c\rangle V$ we can simplify $\langle \delta^2 \langle f|V\rangle\rangle$ into

$$\langle \delta^2 \langle f|V\rangle_f\rangle_V = \langle \delta^2 \langle c\rangle V\rangle_V = \langle c\rangle^2 \langle \delta^2 V\rangle_V \tag{13}$$

and since $\langle f\rangle = \langle c\rangle\langle V\rangle$ we can rewrite the noise term $\frac{\langle \delta^2 \langle f|V\rangle\rangle}{\langle f\rangle^2}$ as

$$\frac{\langle \delta^2 \langle f|V\rangle_f\rangle_V}{\langle f\rangle^2} = \frac{\langle \delta^2 V\rangle}{\langle V\rangle^2}, \tag{14}$$

which shows that the noise in total cell fluorescence due to cell growth equals the noise in cell volume. This is due to the fact that total cell fluorescence varies across cells with different volume, which is due to the different cell cycle progression stages of those cells and because the copy number of a constitutively expressed protein scales with cell volume at balanced growth.

Deviations from the dependency between fluorescence per cell, f, and cell volume, V, also occurring at balanced growth, also contribute to noise in total cell fluorescence. Those are quantified by the term,

$$\frac{\langle\langle \delta^2 f|V\rangle_f\rangle_V}{\langle f\rangle^2}, \tag{15}$$

which captures all remaining noise sources: biochemical reactions (e.g. transcription, translation, noise propagation in reaction networks), asymmetric cell division, uneven protein partitioning, etc. [1]. Variance decomposition can also be used to decompose this term further, as was shown in Schwabe & Bruggeman [1]. Note that only in the simplest cases, the Poisson relation holds that $\frac{\langle\langle \delta^2 f|V\rangle_f\rangle_V}{\langle f\rangle^2} \propto \frac{1}{\langle f\rangle}$.

Simplifying relations for noise in total cell fluorescence at balanced growth

Variance decomposition, with respect to cell volume, leads to the following equation for the noise in fluorescence concentration,

$$\frac{\langle \delta^2 c\rangle}{\langle c\rangle^2} = \frac{\langle\langle \delta^2 c|V\rangle_c\rangle_V}{\langle c\rangle^2} + \frac{\langle \delta^2 \langle c|V\rangle_c\rangle_V}{\langle c\rangle^2}. \tag{16}$$

At balanced growth the simplification holds that

$$\langle c|V\rangle = \frac{\langle f|V\rangle}{V} = \frac{\langle c\rangle V}{V} = \langle c\rangle \quad \Rightarrow \quad \langle \delta^2 \langle c|V\rangle_c\rangle_V = 0, \tag{17}$$

therefore, at balanced growth,

$$\frac{\langle \delta^2 c\rangle}{\langle c\rangle^2} = \frac{\langle\langle \delta^2 c|V\rangle_c\rangle_V}{\langle c\rangle^2}. \tag{18}$$

Noise in fluorescence concentration and its variance decomposition at balanced growth

The noise in a ratio of random variables, such as the concentration f/V, can be approximated by

$$\begin{aligned} \frac{\langle \delta^2 c\rangle}{\langle c\rangle^2} &= \frac{\langle \delta^2 \frac{f}{V}\rangle}{\langle \frac{f}{V}\rangle^2} \approx \frac{\langle \delta^2 f\rangle}{\langle f\rangle^2} - 2\frac{\langle \delta f \delta V\rangle}{\langle f\rangle\langle V\rangle} + \frac{\langle \delta^2 V\rangle}{\langle V\rangle^2} \\ &= \frac{\langle\langle \delta^2 f|V\rangle_f\rangle_V}{\langle f\rangle^2} - 2\frac{\langle \delta f \delta V\rangle}{\langle f\rangle\langle V\rangle} + 2\frac{\langle \delta^2 V\rangle}{\langle V\rangle^2} \end{aligned} \tag{19}$$

The covariance between total cell fluorescence and cell volume $\langle \delta f \delta V\rangle$ can be simplified into, using $f|V = \langle c\rangle V + \epsilon(V)$ with $\epsilon(V) \sim N(0, \sqrt{\langle \delta^2 f|V\rangle})$,

$$\begin{aligned} \langle \delta f \delta V\rangle &= \langle (f - \langle f\rangle)(V - \langle V\rangle)\rangle = \langle (\langle c\rangle V + \epsilon(V) - \langle c\rangle\langle V\rangle)(V - \langle V\rangle)\rangle \\ &= \langle \langle c\rangle V^2 - \langle c\rangle V\langle V\rangle + \epsilon(V)V - \epsilon(V)\langle V\rangle - \langle c\rangle\langle V\rangle V + \langle c\rangle\langle V\rangle^2\rangle \\ &= \langle c\rangle\langle V^2\rangle - \langle c\rangle\langle V\rangle^2 + \langle \epsilon(V)V\rangle - \langle \epsilon(V)\rangle\langle V\rangle - \langle c\rangle\langle V\rangle^2 + \langle c\rangle\langle V\rangle^2 \\ &= \langle c\rangle\langle \delta^2 V\rangle + \langle \delta\epsilon(V)\delta V\rangle \end{aligned} \tag{20}$$

such that

$$\frac{\langle \delta f \delta V\rangle}{\langle f\rangle\langle V\rangle} = \frac{\langle c\rangle\langle \delta^2 V\rangle + \langle \delta\epsilon(V)\delta V\rangle}{\langle c\rangle\langle V\rangle^2} = \frac{\langle \delta^2 V\rangle}{\langle V\rangle^2} + \frac{\langle \delta\epsilon(V)\delta V\rangle}{\langle c\rangle\langle V\rangle^2}. \tag{21}$$

This simplifies equation 19 into

$$\frac{\langle \delta^2 c\rangle}{\langle c\rangle^2} = \frac{\langle\langle \delta^2 f|V\rangle_f\rangle_V}{\langle f\rangle^2} - 2\frac{\langle \delta\epsilon(V)\delta V\rangle}{\langle c\rangle\langle V\rangle^2}. \tag{22}$$

A relation between noise in total cell fluorescence and fluorescence concentration

Therefore, if the noise in fluorescence at a fixed volume, $\epsilon(V)$, is volume independent, such that $\langle \delta\epsilon(V)\delta V\rangle = 0$, we obtain

$$\frac{\langle \delta^2 c\rangle}{\langle c\rangle^2} = \frac{\langle\langle \delta^2 f|V\rangle_f\rangle_V}{\langle f\rangle^2} = \frac{\langle\langle \delta^2 c|V\rangle_c\rangle_V}{\langle c\rangle^2} := \text{ protein-expression noise.} \tag{23}$$

This equation indicates why it is advantageous to focus on noise in fluorescence concentration, rather than on noise in total cell fluorescence, as it only captures the noise effects due to the biochemistry of the circuit and cellular processes such as asymmetric division and uneven protein partitioning. It is therefore independent of the heterogeneity in protein expression due to the fact that total cell fluorescence scales with volume, due to cell growth. The latter effect is captured by $\frac{\langle \delta^2 \langle f|V\rangle_f\rangle_V}{\langle f\rangle^2}$ and equals $\frac{\langle \delta^2 V\rangle}{\langle V\rangle^2}$ at balanced growth.

3 Supplementary figures

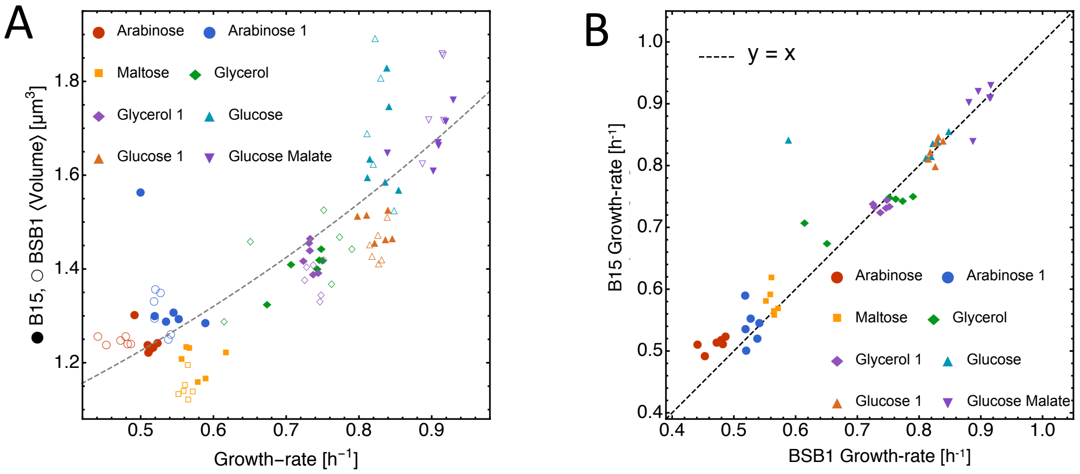

Figure 1: **Average cell volume increases with growth rate. Protein burden from GFP expression is negligible. A.** Average cell volume increases as function of growth rate. For each experiment, volumes were averaged over the range of balanced growth. Each data point represents a different IPTG concentration under a certain growth condition. Symbol shape indicates carbon source as indicated in the legend with numbers denoting biological replicates. Filled symbols, *B. subtilis* B15 with titratable GFP inserted in the *amyE* locus. Empty symbols, *B. subtilis* BSB1 wild-type. **B.** Growth rate of BSB1 wild-type and B15 GFP expressing mutant are plotted against each other. A decrease in growth rate due to protein burden from expressing GFP was not detected.

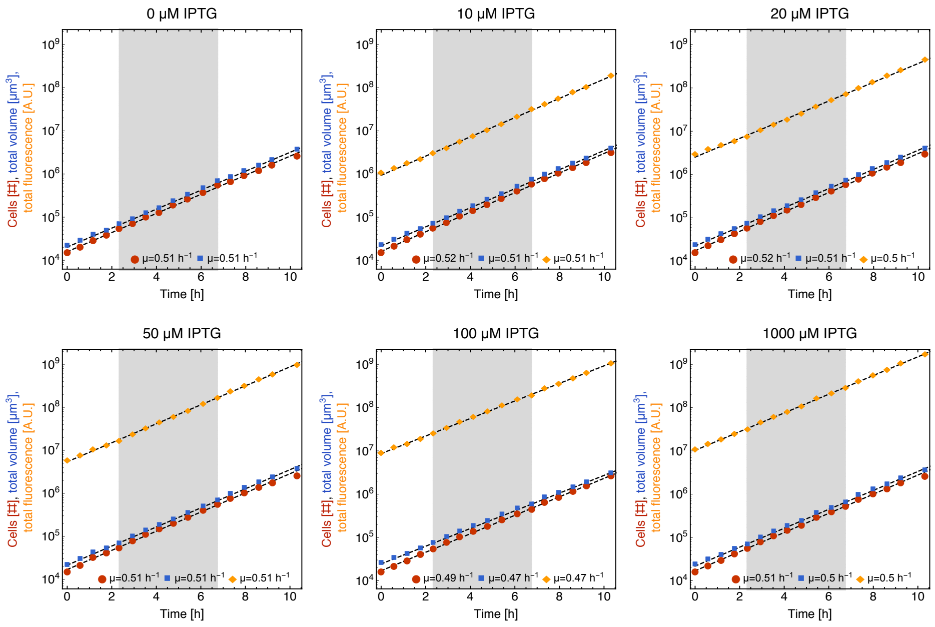

Figure 2: **Balanced growth of *B. subtilis* B15 on arabinose, sample A at different IPTG concentrations.** Population volume and -fluorescence increase at the same rate as cell numbers, indicating balanced growth. The dashed lines indicate linear fits whose slope equals the specific growth rate that is indicated at the bottom of the plot for each property (number of cells, population volume and - fluorescence). The grey background marks the region in which fluorescence concentration was most stable. Data points from this region where used for all analyses in this study. Capital letters (A,B) indicate biological replicates.

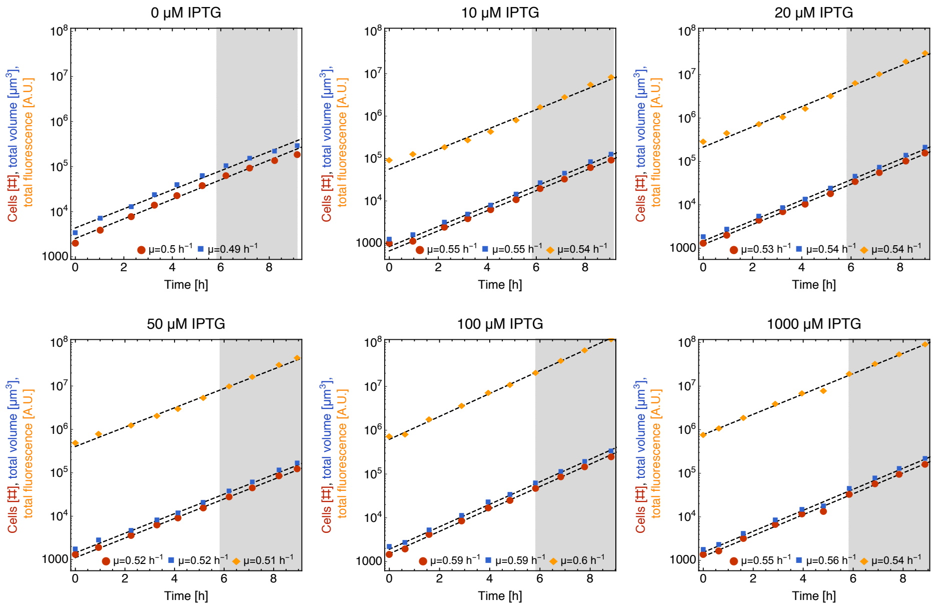

Figure 3: **Balanced growth of *B. subtilis* B15 on arabinose, sample B at different IPTG concentrations.** See figure 11 for further detail.

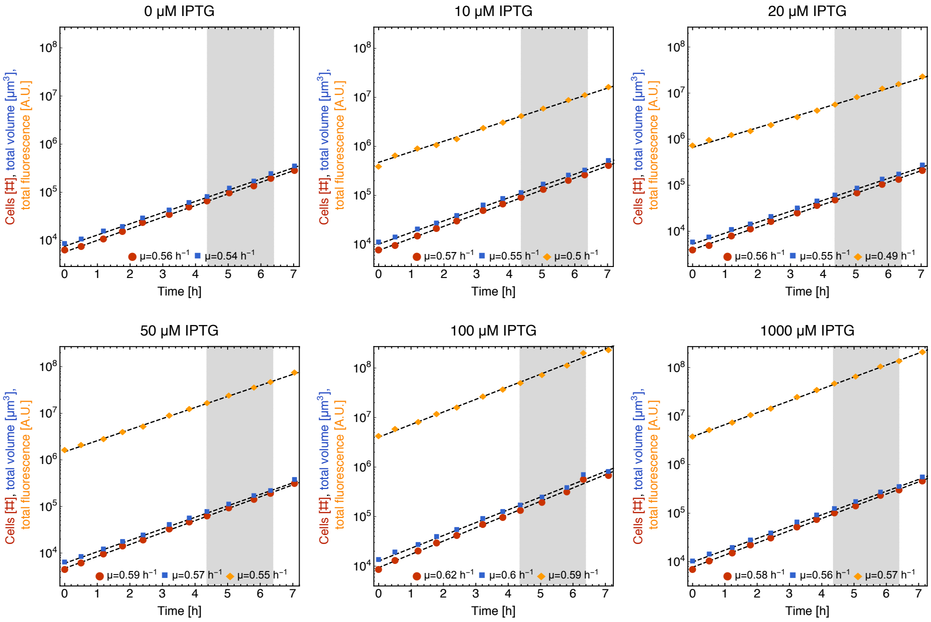

Figure 4: **Balanced growth of *B. subtilis* B15 on maltose at different IPTG concentrations.** See figure 11 for further detail.

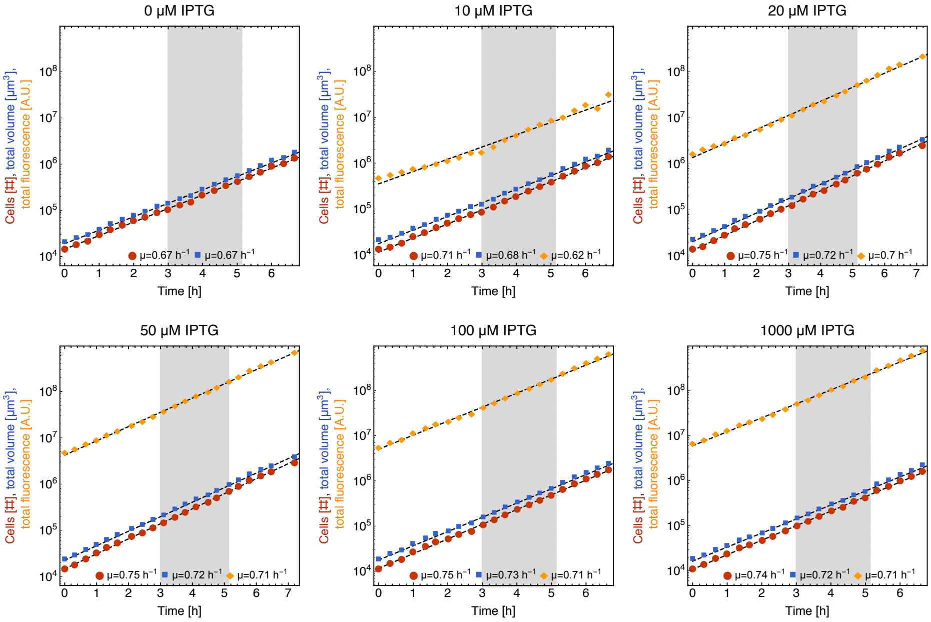

Figure 5: **Balanced growth of *B. subtilis* B15 on glycerol, sample A at different IPTG concentrations.** See figure 11 for further detail.

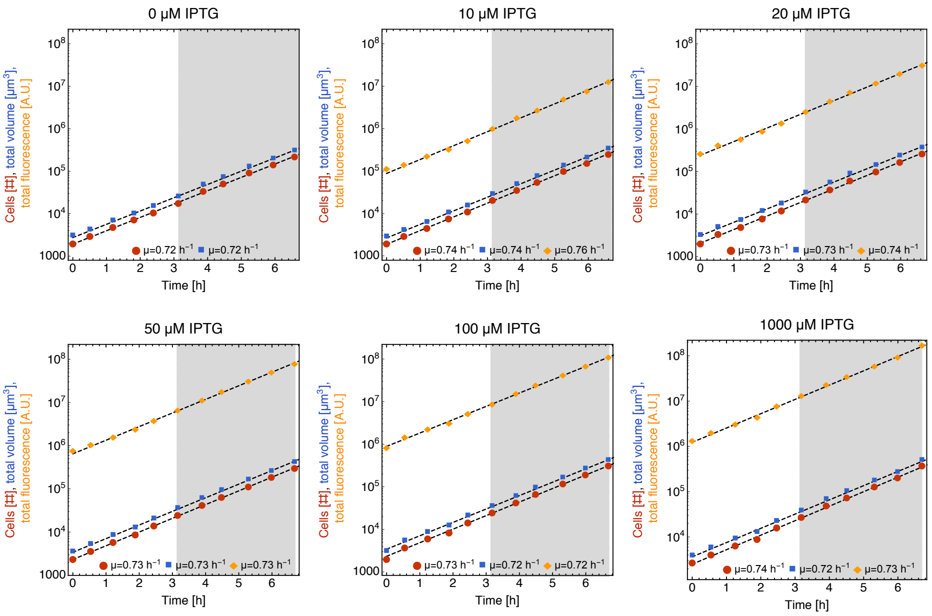

Figure 6: **Balanced growth of *B. subtilis* B15 on glycerol, sample B at different IPTG concentrations.** See figure 11 for further detail.

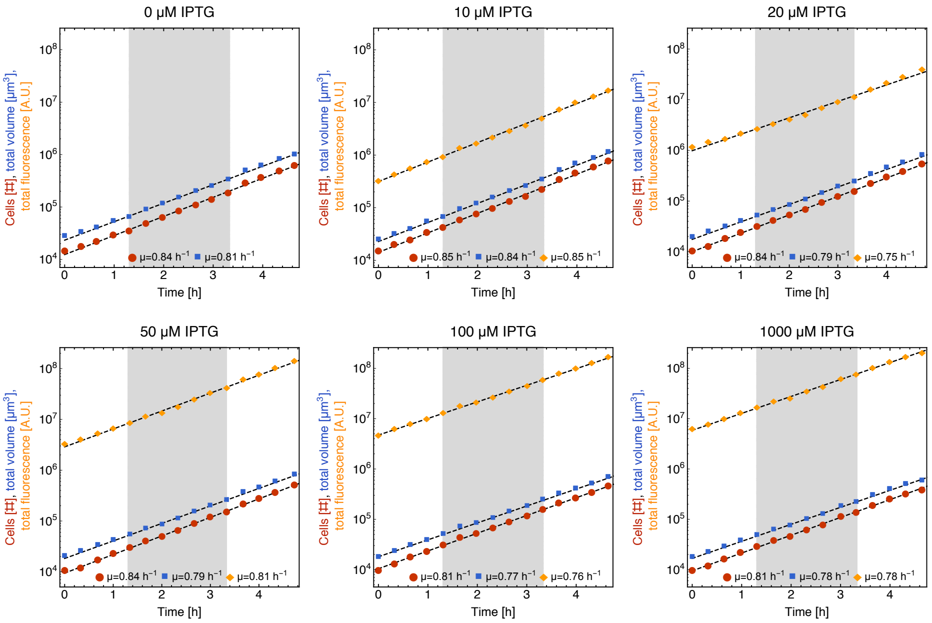

Figure 7: **Balanced growth of *B. subtilis* B15 on glucose, sample A at different IPTG concentrations.** See figure 11 for further detail.

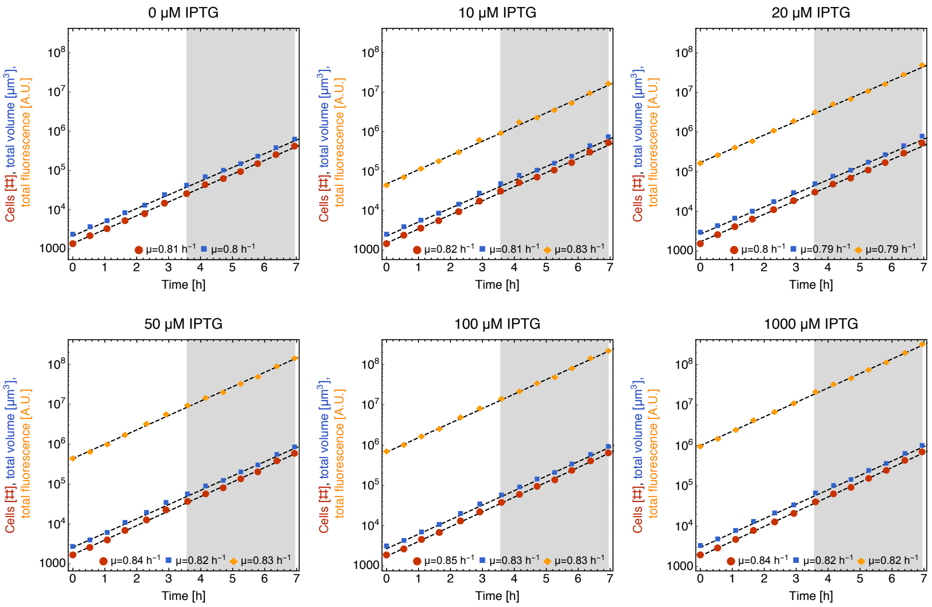

Figure 8: **Balanced growth of *B. subtilis* B15 on glucose, sample B at different IPTG concentrations.** See figure 11 for further detail.

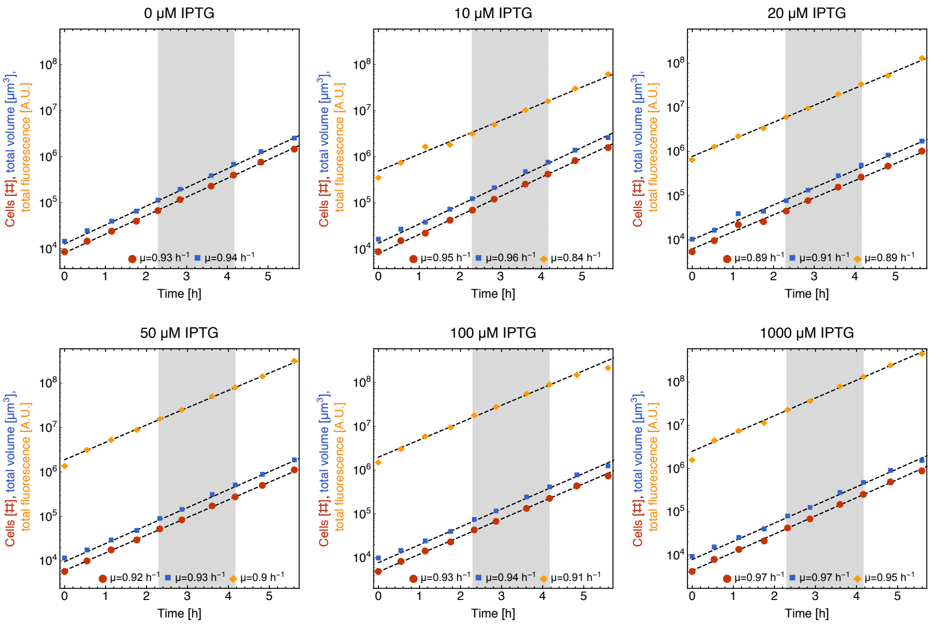

Figure 9: **Balanced growth of *B. subtilis* B15 on glucose + malate at different IPTG concentrations.** See figure 11 for further detail.

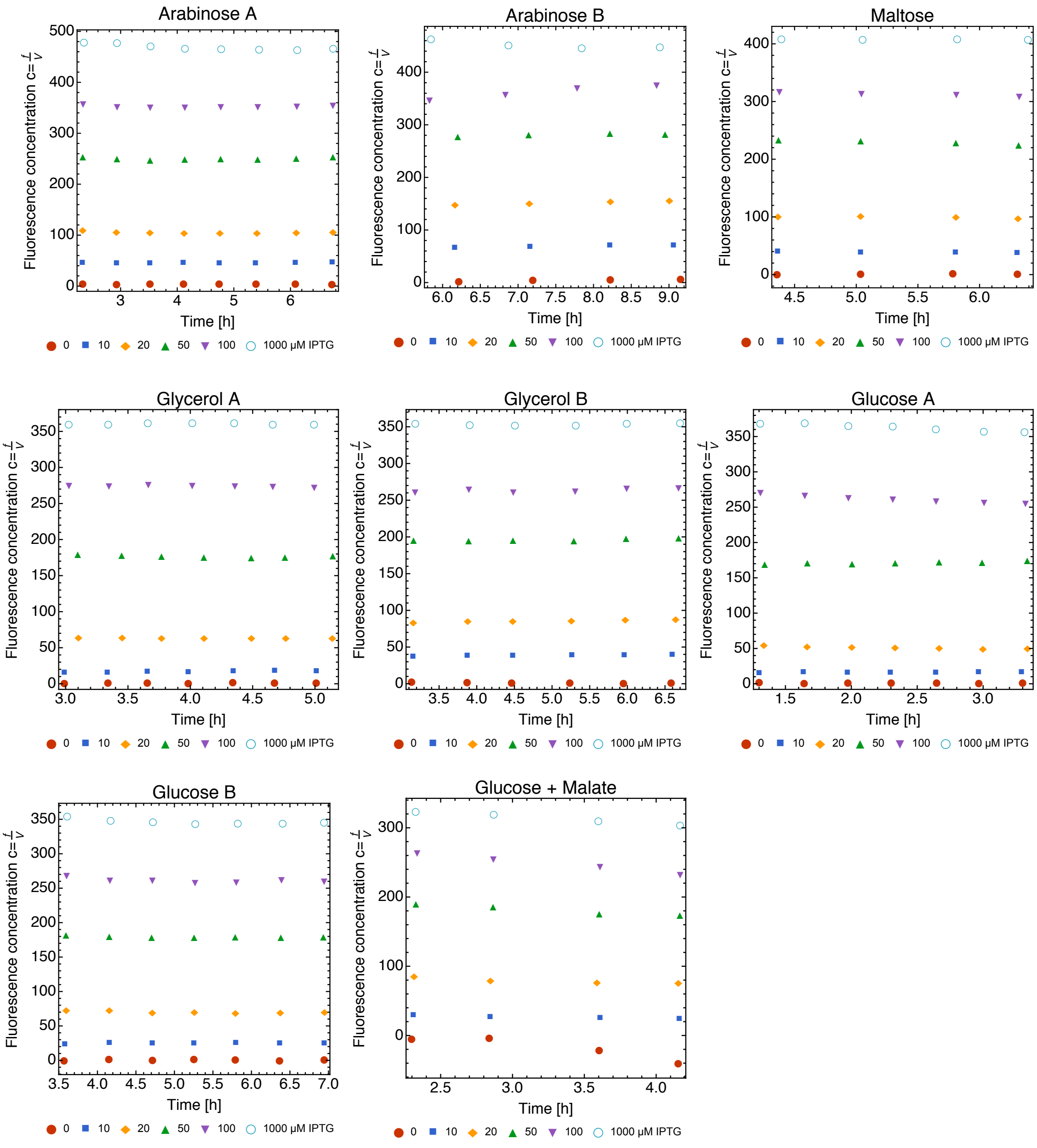

Figure 10: **Concentration homeostasis during balanced growth.** For each experiment, the region of balanced growth is shown (cmp. Figures S2-9). Capital letters (A,B) indicate biological replicates. Per experiment, the average of all time points from the region of balanced growth was used for further analyses.

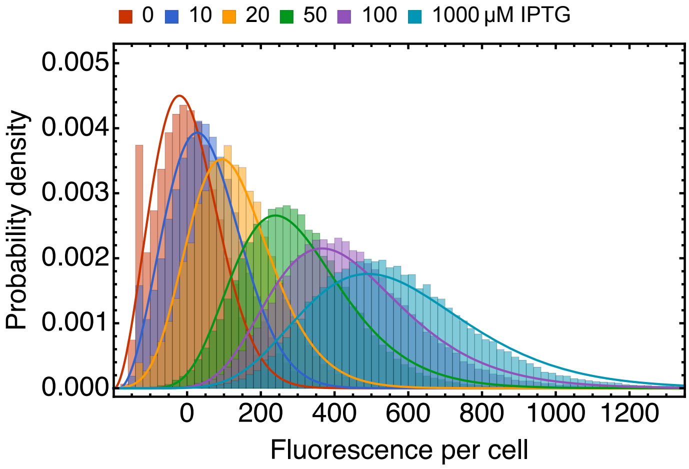

Figure 11: **Distributions of fluorescence per cell of *B. subtilis* B15 growing on arabinose at different IPTG concentrations.** This figure accompanies Figure 2b in the main text. The measured distributions are fitted to gamma distributions (solid lines).

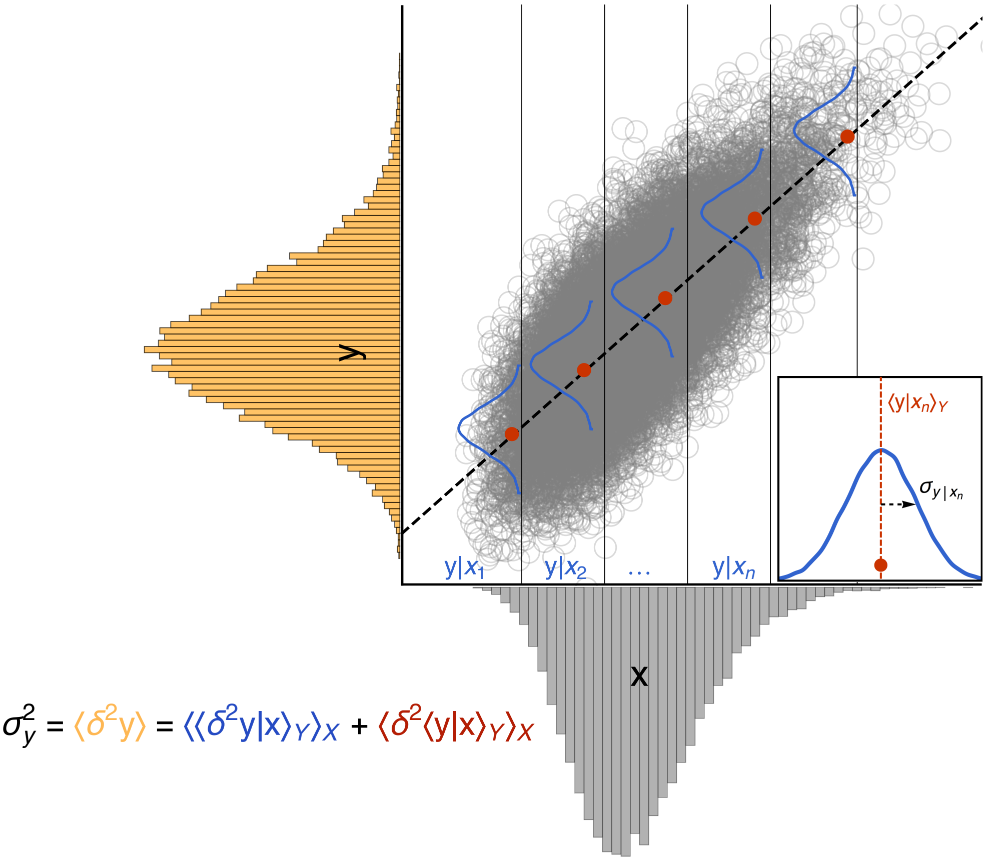

Figure 12: **Visualisation of the law of total variance.** The random variables X and Y are distributed according to the marginal distributions in gray and yellow, respectively. The values of Y depend on X by a function $f(x) = \langle y|x \rangle$ as indicated by the dashed line. At fixed x_n (inset), the values of Y follow a distribution (blue) with a mean, indicated in red, and a standard deviation $\sigma_{y|x_n}$, indicated by the black arrow. The variance of Y ($\langle \delta^2 y \rangle$, marked in yellow) is the sum of 1.) the mean variance of the conditional distributions ($\langle \langle \delta^2 y|x \rangle_Y \rangle_X$, marked in blue) and 2.) the variance of the conditional means ($\langle \delta^2 \langle y|x \rangle_Y \rangle_X$, marked in red). Variations in $y|x$, captured by $\langle \langle \delta^2 y|x \rangle_Y \rangle_X$, are solely due to fluctuations in y that are intrinsic to y and independent of x, whereas the variations in $\langle y|x \rangle$, given by $\langle \langle \delta^2 y|x \rangle_Y \rangle_X$, are extrinsic to y and can be attributed to changes in x. When X and Y are independent, the latter term becomes zero (which is the case for concentration at balanced growth).

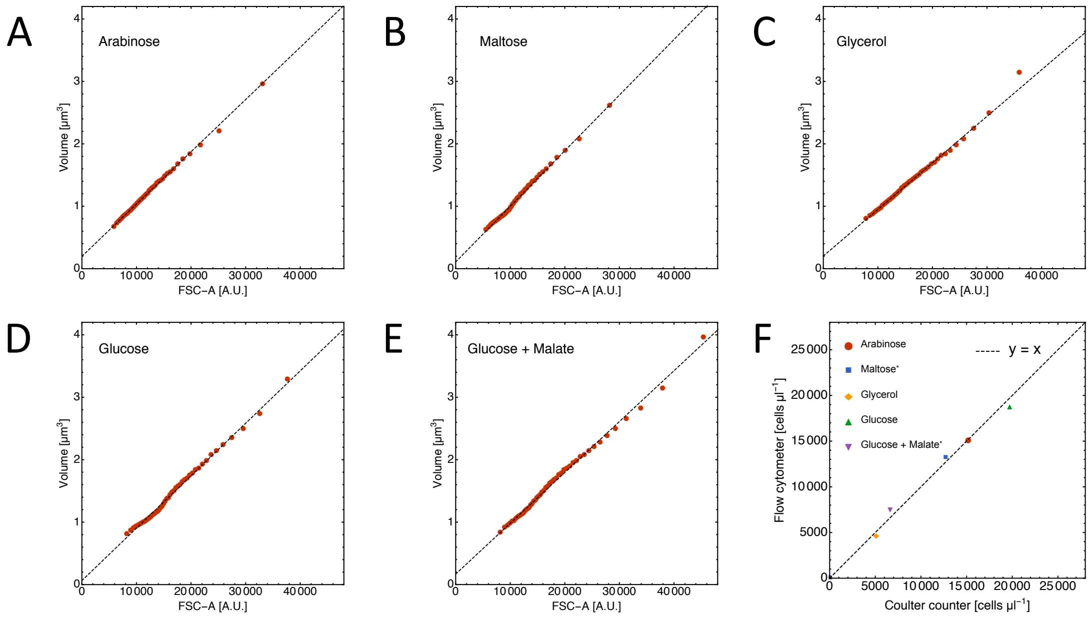

Figure 13: **Cross calibration of forward scatter to volume. A-E.** Cross-calibration of forward scatter area (FSC-A) to volume. Data points indicate quantiles in FSC-A and volume distributions mapped against each other. Dashed lines indicate the individual linear fits that are used to convert FSC-A to volume. For further detail, refer to the Methods (main text). **F.** After correcting for dilution, flow cytometer and Coulter counter registered the same number of cells per sample.

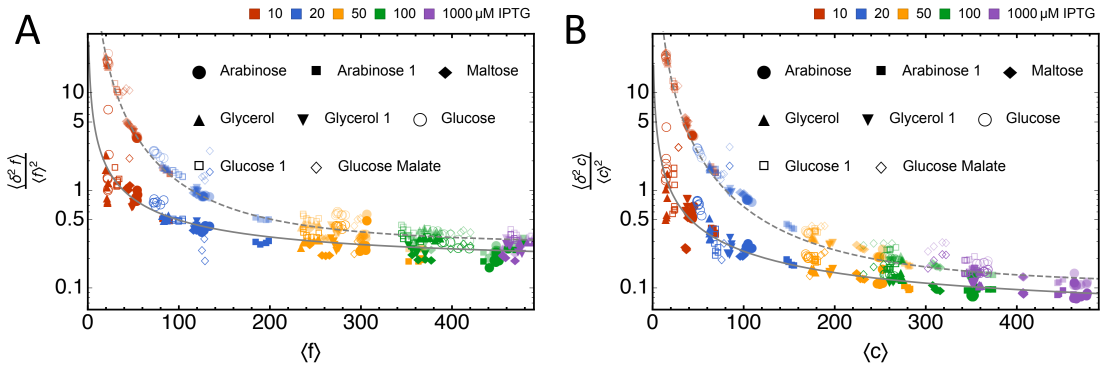

Figure 14: **Variance of background fluorescence distorts the scaling of noise with mean expression levels.** Noise in total cell fluorescence (A.) and fluorescence concentration (B.) as function of the respective mean across growth rates and promoter activities (i.e. IPTG concentrations), with (dense symbols) and without (light symbols) corrections for the variance of background fluorescence. Solid and dashed lines indicate $\frac{\langle\delta^2 x\rangle}{\langle x\rangle^2} \propto \frac{a}{\langle x\rangle}$ and $\frac{\langle\delta^2 x\rangle}{\langle x\rangle^2} \propto \frac{\langle\langle\delta^2 x_{bg}\rangle\rangle}{\langle x\rangle^2}$, respectively. The solid lines are the same as in figure 4, main text. In both plots, all time points from the region of balanced growth are shown (see figure 10).

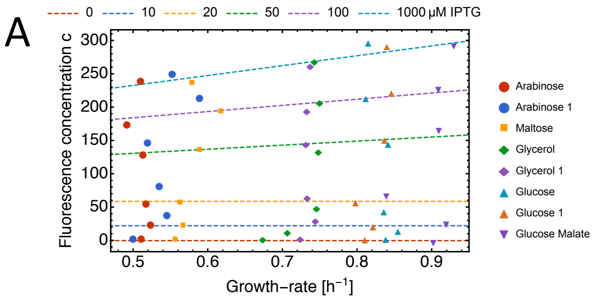

Figure 15: **Reduced protein expression by dilution by growth is partially compensated for by enhanced protein synthesis rate at higher growth rates.** Supplemental figure to plot 3 BC, of the main text, which shows all individual experiments. Each data point is the average of all time points shown in figure 10. Dashed lines, fits as in figure 3 of the main text.

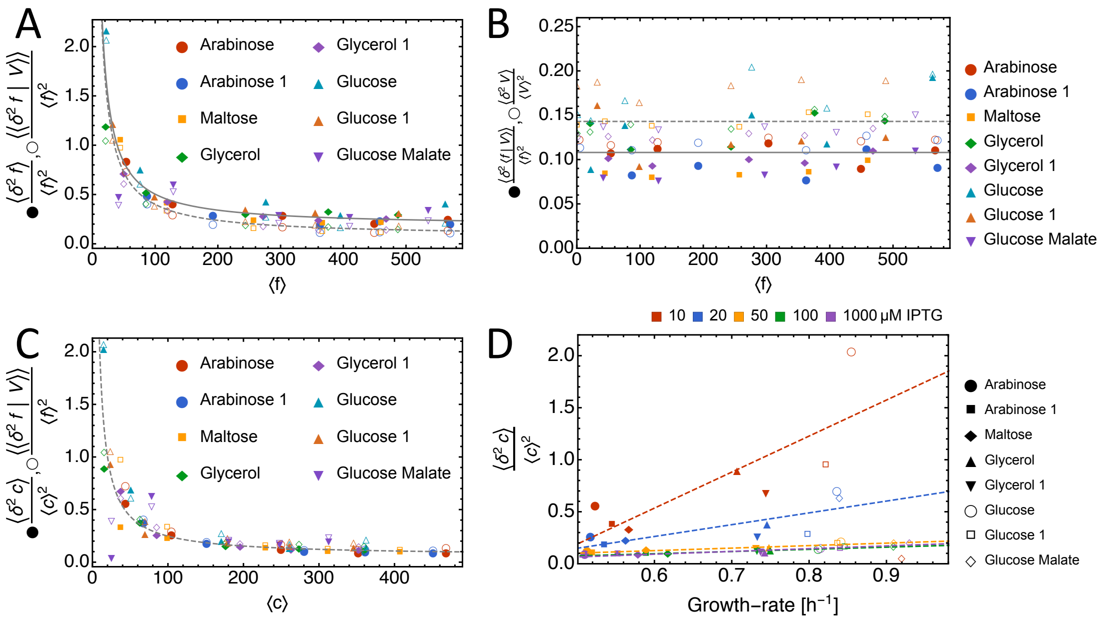

Figure 16: **Effects of promoter activity and growth rate on noise are indistinguishable and fall on an invariant relation between noise and mean of protein expression.** Supplemental figure to figure 4, main text, showing the results of all individual experiments. **A.** Noise in total cell fluorescence (filled symbols) and its intrinsic, 'biochemical' contribution $\frac{\langle\langle \delta^2 f|V\rangle\rangle}{\langle f\rangle^2}$ (open symbols). **B.** Fluorescence variation scales with volume as indicated by the relation $\frac{\langle \delta^2\langle f|V\rangle\rangle}{\langle f\rangle^2} = \frac{\langle \delta^2 V\rangle}{\langle V\rangle^2}$ which holds at balanced growth. From our experimental data, we see that $\frac{\langle \delta^2\langle f|V\rangle\rangle}{\langle f\rangle^2} \approx \frac{\langle \delta^2 V\rangle}{\langle V\rangle^2}$. **C.** At balanced growth, noise in fluorescence concentration directly captures protein expression noise due to biochemical origins (i.e. intrinsic sources). **D.** Dependence of noise in fluorescence concentration on the cellular growth rate at different magnitudes of promoter activity (IPTG concentration). For each carbon source, data points from 10 to 1000 μM IPTG are shown. Each data point is the average of all time points at balanced growth shown in figure 10.

References

[1] Schwabe, A. and Bruggeman, F. J. (jul, 2014) Contributions of cell growth and biochemical reactions to nongenetic variability of cells. *Biophysical Journal,* **107**(2), 301–313.
